# Flash Communication: Arene η^6^‑Coordination to Chromium Tricarbonyl Promotes Cobalt-Catalyzed C–H Borylation of Electron-Rich Arenes

**DOI:** 10.1021/acs.organomet.6c00233

**Published:** 2026-08-25

**Authors:** Richard D. Broene, Lauren Sablone, Tianyi Zhang, Carlota Odena, Alex M. Shimozono, Paul J. Chirik

**Affiliations:** Department of Chemistry, 6740 Princeton University, Princeton, New Jersey 08544, United States

## Abstract

The cobalt-catalyzed borylation of electron-rich arenes η^6^-coordinated to [Cr­(CO)_3_] has been demonstrated using the pyridine­(dicarbene) pincer-supported dicobalt alkoxide (^iPr^ACNC)­Co_2_(^t^BuO)_4_ as the precatalyst. Metal coordination accelerated the borylation of the arene by 20−200 fold, enabling reactivity with substrates that were otherwise sluggish or inactive for cobalt-catalyzed C–H functionalization. The cobalt-catalyzed borylation of η^6^-coordinated electron-rich arenes proceeded at ambient temperature in the absence of HBPin scavengers. Arene coordination to chromium also proved to be an effective strategy for reversal of the traditional chemoselectivity of metal-catalyzed C­(sp^2^)–H borylation as 2-fluorobiphenyl was exclusively functionalized at the more electron-rich ring.

## Graphical abstract

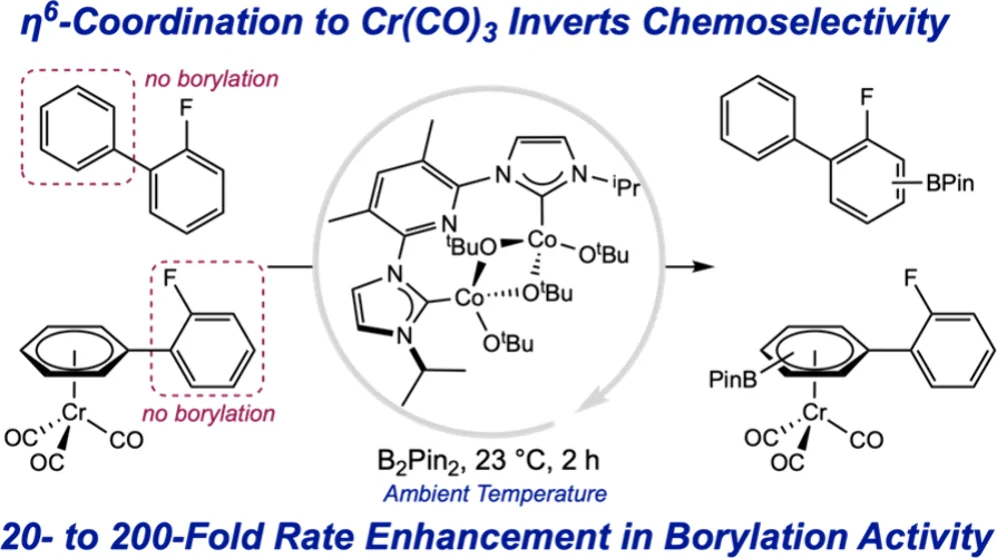

The site-selective borylation of (hetero)­arene C­(sp^2^)–H bonds has emerged as one of the most widely applied methods of C–H functionalization, owing to the availability of high-activity catalysts, predictable site selectivity and the versatility of the resulting C–B bond in organic synthesis.^1^ Cobalt precatalysts have been developed as alternatives to well-established, state-of-the-art iridium catalysts^2^ and offer distinct site selectivity oftentimes based on the electronic properties of various C–H bonds (Scheme 1A).^3–7^ Among the various catalyst types, dicobalt alkoxide complexes bearing alkylimidazole-substituted pyridine dicarbene (^R^ACNC) pincers have recently been reported that are straightforward to synthesize and active for the site selective borylation of a range of arenes, including late-stage pharmaceutical intermediates.^8^

**Scheme 1:**
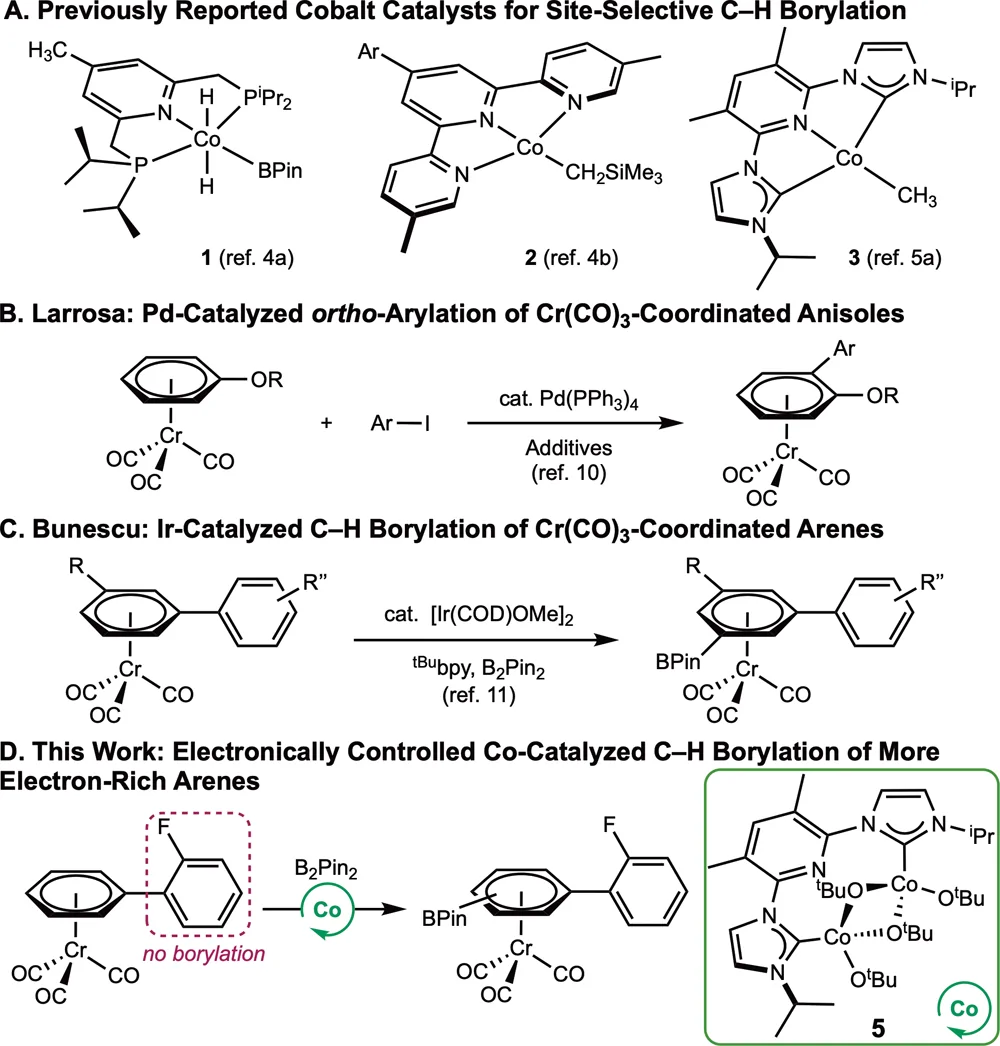
Cobalt Precatalysts for C–H Borylation and Coordination of Arenes to [Cr­(CO)_3_] as a Strategy to Accelerate Metal-Catalyzed C­(sp^2^)–H Functionalization

Despite these advances, the borylation of electron-rich arenes remains challenging, in part due to the higher barriers associated with C–H oxidative addition. Relatedly, achieving site-selective borylation of electron-rich aromatic rings in the presence of more activated, electron-deficient arenes is difficult as the latter undergo C–H oxidative addition at substantially faster rates. One strategy to overcome these reactivity differences is coordination of the arene to an appropriate transition metal.^9^ Larrosa and co-workers pioneered a method for the activation of substituted anisoles for selective Pd-catalyzed arylation by π-complexation of aromatic ring with chromium tricarbonyl [Cr­(CO)_3_] (Scheme 1B).^10^ This enhanced the rate of C–H functionalization by up to 4 orders of magnitude over reactions with the free arene and provided high yields of ortho-arylated anisole derivatives. Substrate coordination to [Cr­(CO)_3_] reduces electron density in the π-system and enhances C–H out-of-plane bending, which facilitates a concerted metalation-deprotonation pathway previously unavailable to relatively electron-rich arenes. During the course of our studies, Bunescu and co-workers applied this strategy to iridium-catalyzed C–H borylation reactions (Scheme 1C), giving rise to both mono- and polyaromatic borylated products in excellent yields.^11a^ DFT studies support a hypothesis where the barrier for C–H oxidative addition is lower with the substrate-bound Cr­(CO)_3_-coordination complex. More recent studies using tailored iridium and cobalt catalysts have also been reported by the same group.^11b,11c^ Ilies and co-workers have also reported that the combination of Co­(acac)_2_ with various terpyridine ligands is effective for the borylation of (η^6^-arene)­Cr­(CO)_3_ complexes with B_2_(EPin)_2_, including electron-rich substrates.^12^

Given the substantial rate enhancements observed in C–H functionalization of arenes coordinated to [Cr­(CO)_3_] and the electronically driven site selectivity observed with pincer-supported cobalt catalysts, application of this strategy using (^iPr^ACNC)­Co_2_(^t^BuO)_4_ as the precatalyst was explored. One of the goals of this work is to invert the chemoselectivity of site-selective borylation where an electron-rich ring is functionalized over an electron poor one (Scheme 1D).^13,14^ Our studies commenced with the preparation of (1,3-di-*tert*-butylbenzene)­Cr­(CO)_3_ (**4a**) by refluxing one equivalent of the arene with 1.3 equiv of Cr­(CO)_6_ in a 10:1 mixture of ^n^Bu_2_O and THF.^9,15^ Catalytic borylation of the complex was conducted with 5 mol % of (^iPr^ACNC)­Co_2_(^t^BuO)_4_ and 1.1 equiv of B_2_Pin_2_ in THF solution (Scheme 2). After 2 h at ambient temperature, the borylated piano-stool complex **4b** was formed in 86% assay yield by NMR spectroscopy with exclusive selectivity for the sterically accessible C­(sp^2^)–H bond. Notably, at longer reaction times of 24 h, the free borylated arene was observed arising from dissociation of the hindered product from the chromium. In a control experiment, no evidence for borylation was obtained by NMR spectroscopy when **4a** was treated with 1.1 equiv of B_2_Pin_2_ over extended periods of time in the absence of the cobalt precatalyst, **5**.^12b,16^

**Scheme 2:**
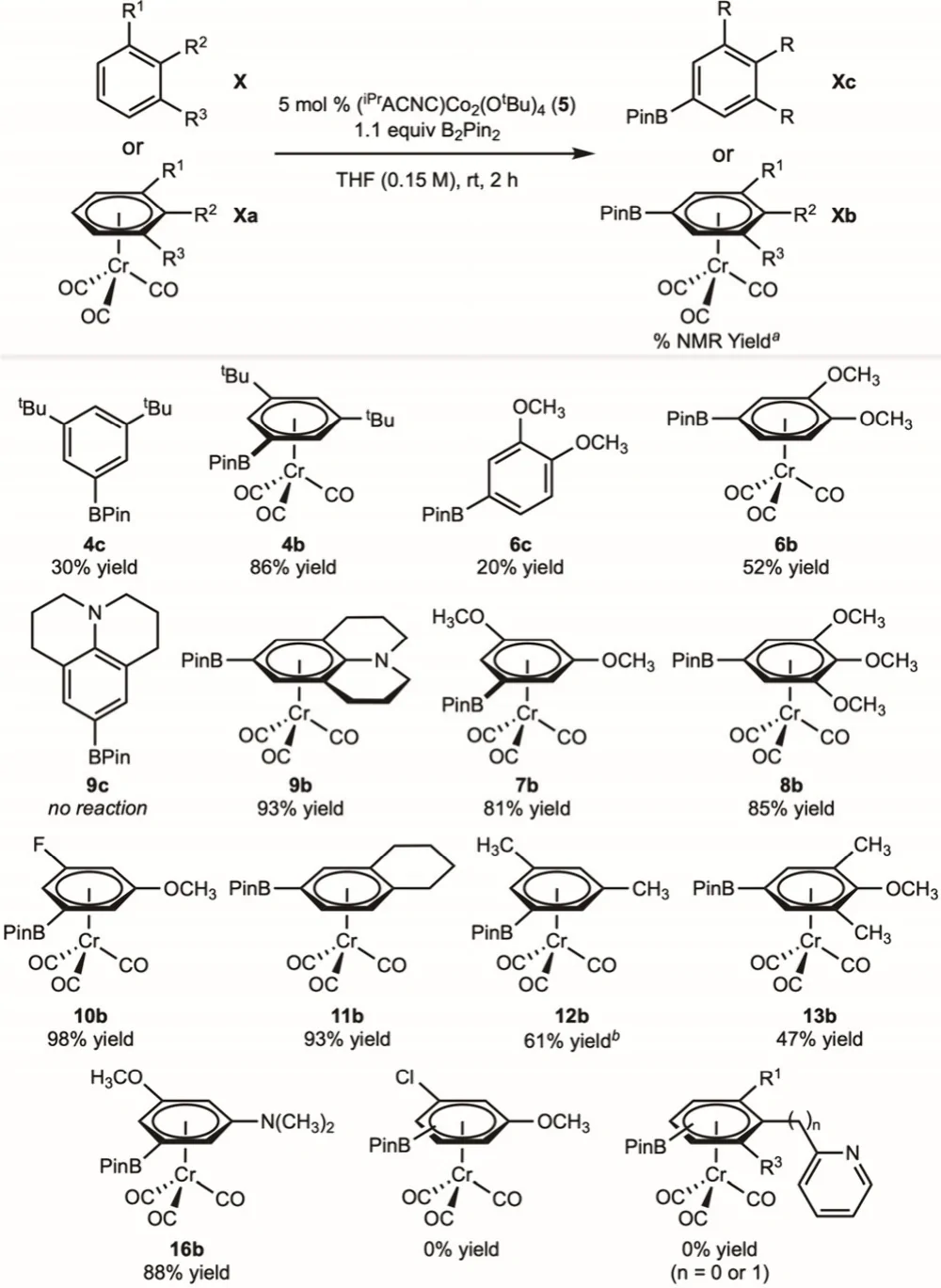
Catalytic C­(sp^2^)–H Borylation of Electron-Rich Arenes in the Presence and Absence of Coordination to [Cr­(CO)_3_] using (^iPr^ACNC)­Co_2_(O^t^Bu)_4_ (**5**) as the Precatalyst

a Relative to the 1,3,5-trimethoxybenzene internal standard.
b Additional benzylic borylation was also observed.

The cobalt-catalyzed method was explored with other arenes that, as free substrates, were challenging for borylation. Under standard conditions, the chromium carbonyl complex of veratrole (**6a**) underwent borylation in 52% yield with exclusive selectivity for the sterically accessible position as compared to 20% for free **6**. With the η^6^-1,3-dimethoxybenzene complex **7a**, product **7b** was obtained in 81% yield and with η^6^-1,2,3-trimethoxybenzene complex **8a** the borylated product **8b** was observed in 85% yield. Julolidine **9**, which failed to borylate under the previously reported conditions,^8^ furnished 93% yield of **9b**, when the chromium tricarbonyl complex **9a** was used at ambient temperature. In comparison, the state of the art in iridium-catalyzed C–H borylation of free arene **9** requires heating at 80 °C for 16 to 24 h.^17^ Both 1,2- and 1,3-disubstituted and 1,2,3-trisubstituted arenes (**10**, **11** and **13**) afforded products resulting from borylation at the sterically less hindered position in good to excellent yields (47–98%). For these electron-rich substrates, the sterically favored *meta* product is typically unavailable by classical electrophilic substitution chemistry, and the current route provides products with otherwise challenging to access arene substitution patterns. Moreover, arene coordination to [Cr­(CO)_3_] obviates the need for olefin additives such as *tert*-butylethylene as HBPin scavengers, while preserving the electronically driven site selectivity of the cobalt catalyst.^8^ For example, catalytic borylation of the (η^6^-3-fluoroanisole)­Cr­(CO)_3_ complex **10a** resulted in high yield of the same *meta*-to-F borylated product **10b** previously reported using **5**.^8^

Attempts to expand the scope of the borylation reaction to include aryl halides beyond fluoroarenes did not yield borylated products, likely due to catalyst deactivation by C–X oxidative addition to the cobalt­(I) active species.^11^ Additionally, pyridine-containing substrates were resistant to borylation, with only trace yields of products observed even at extended reaction times. Use of an in situ catalyst generation method whereby prestirring **5** with B_2_Pin_2_ for 1 h at ambient temperature followed by addition of the arene-supported chromium complex did not result in improved conversions. As was observed in reactions in the absence of chromium coordination,^14^ methylated arenes such as **12a** showed further borylation at benzylic C­(sp^3^)–H position.

Arene substrates were explored where coordination to the chromium fragment offered the potential to invert the typical chemoselectivity observed in metal-catalyzed C­(sp^2^)–H borylation. For example, free 2-fluorobiphenyl (**14**) underwent borylation with 5 mol % of (^iPr^ACNC)­Co­(CH_3_) (**3**) to provide a 55% combined yield of *meta*- and *ortho*-to-fluorine borylated products in an 83:17 ratio, respectively (Scheme 3A­(i)).^8^ Coordination of this substrate to chromium was selective for the more-electron rich ring to yield **14a**. Subjecting this compound to standard catalytic conditions provided a 78% combined yield of *meta*, *para* and *bis-meta* substituted products in a 19:37:44 ratio (Scheme 3A­(ii)). Importantly, no borylation was detected at the fluorinated ring.

**Scheme 3:**
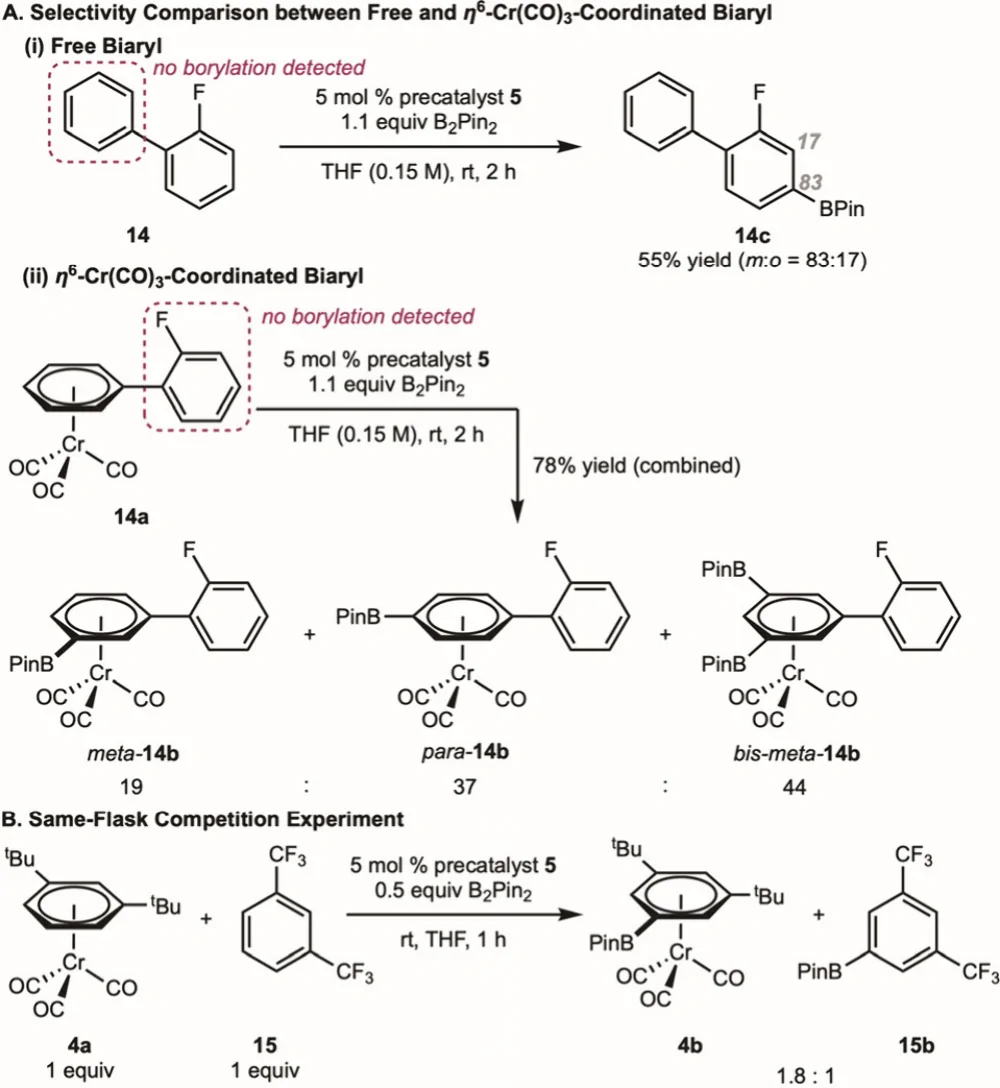
Comparison of Reactivity and Site Selectivity in Co-Catalyzed C–H Borylation of Free and Cr-Coordinated Arene Substrates^a^

a NMR assay yields were measured relative to those of 1,3,5-trimethoxybenzene as an internal standard.

Additional experiments demonstrated that the chromium-coordinated, electron-rich arenes underwent more rapid cobalt-catalyzed C­(sp^2^)–H borylation than free, electron-deficient ones. Catalytic borylation of an equimolar quantity of (η^6^-1,3-(^t^Bu)_2_-C_6_H_4_)­Cr­(CO)_3_ **4a** and 1,3-(CF_3_)_2_-C_6_H_4_ (**15**) with 5 mol % of **5** in the presence of 0.5 equiv of B_2_Pin_2_ were stirred for 1 h at ambient temperature and provided 1.8:1 ratio of **4b** to **15b** (Scheme 3B). Notably, the borylation of an equimolar mixture of free 1,3-(^t^Bu)_2_-C_6_H_4_ (**4**) and **15** under the same conditions resulted in the borylation of only **15**, with no evidence for reaction with **4**, suggesting a relative rate of borylation of at least 100:1 considering the detection limits of the NMR experiment. Combined with results in Scheme 3B, this indicates that η^6^-coordination of the arene to the [Cr­(CO)_3_] fragment led to a rate acceleration of approximately 200-fold. A separate-flask competition experiment was also conducted between free (**12**) and coordinated (**12a**) *m*-xylene, using 5 mol % of **5** and 1.1 equiv of B_2_Pin_2_ at room temperature for 40 min. A 20-fold rate acceleration was observed by quantitative ^1^H NMR spectroscopy. These results are consistent with the data provided by both Larrosa and Bunescu,^10,11^ who reported substantial rate enhancements, in some cases by several orders of magnitude, in their chromium coordinated arene C–H activation reactions.

These results show that the coordination of arenes to chromium tricarbonyl enhances the rate of ACNC-cobalt catalyzed C–H borylation reactions and extends the ability to control selectivity of the reactions. Because chromium tricarbonyl coordination occurs preferentially for more electron rich rings, complexation offers a route for selectively inverting the chemoselectivity of the cobalt-catalyzed borylation reaction while also expanding its scope. Attempts to render this reaction catalytic in the supporting metal while preserving the distinct site selectivity of these cobalt catalysts are ongoing.

## Supplementary Material



## Notes

General considerations, characterization of borylation products, and competition experiments (PDF)

## †

Department of Chemistry, Bowdoin College, Brunswick, Maine 04011, United States

## Abbreviations

^i^Pr: isopropyl
^t^Bu: *tert*-butyl
B_2_Pin_2_: bis­(pinacolato)­diboron
HBPin: pinacolato borane
B_2_EPin_2_: bis­(1,1,2,2-tetraethylethylene glycolato)­diboron
^R^ACNC: *N*-alkylimidazole pyridine dicarbene ligand.
