# Supplementary material for: Flash Communication: Arene η6‑Coordination to Chromium Tricarbonyl Promotes Cobalt-Catalyzed C–H Borylation of Electron-Rich Arenes
Source: Organometallics. 2026 Aug 25;45(17):2078–82. doi: 10.1021/acs.organomet.6c00233 (PMC13580182; doi:10.1021/acs.organomet.6c00233)
Supplement: Supplementary file 1 [file om6c00233_si_001.pdf]

Supporting Information

Flash Communication: Arene η^6-Coordination to Chromium Tricarbonyl Promotes Cobalt-Catalyzed C–H Borylation of Electron-Rich Arenes

Richard D. Broene, Lauren Sablone, Tianyi Zhang, Carlota Odena, Alex M. Shimozono, and Paul J. Chirik**

Department of Chemistry, Princeton University, Princeton, NJ 08544, U.S.A.

pchirik@princeton.edu

Table of Contents

I. General Considerations S2

II. Preparation of Cobalt Precatalyst S4

III. Preparation of Tricarbonyl Chromium η^6-Arene Complexes S5

IV. General Catalytic Procedures and Products S17

V. Competition Experiment S33

VI. Challenging Substrates S36

VII. References S38

I. General Considerations

All air- and moisture-sensitive manipulations were carried out using vacuum line, Schlenk and cannula techniques or in an MBraun inert atmosphere nitrogen dry box unless otherwise noted. All glassware was stored in a pre-heated oven (≥ 150° C) prior to use. Pentane, hexane, benzene, toluene, diethyl ether, tetrahydrofuran and methylene chloride used for air- and moisture-sensitive manipulations were dried and deoxygenated using literature procedures.[1] Benzene-d_6 used for NMR spectroscopy was distilled from sodium metal and stored over 4 Å molecular sieves. Celite, alumina, and glass fiber filter paper were dried at 180 ºC under vacuum for 3 days prior to use in the glovebox. Solid reagents were dried *in vacuo* overnight while liquid reagents were degassed with at least three freeze/pump/thaw cycles and dried over CaH_2 overnight, before being cycled into an N_2-filled glovebox for general use. Volatile liquids were dried over CaH_2 followed by direct addition through vacuum transfer. Reagents were purchased from Ambeed, Oakwood, Millipore Sigma or TCI America. The following compounds were prepared according to literature procedures: (^{i}PrACNC)Co_2(O^{t}Bu)$_4$,[2] K_2Co_2(O^{t}Bu)$_6$,[3] ^{iPr}ACNC(HBr)$_2$.[4]

^{1}H NMR spectra were recorded on either Bruker AVANCE 300, 400 or 500 or JEOL 400 YH spectrometers operating at 300.13 MHz, 399.8 MHz and 500.46 MHz, respectively. ^{13}C NMR spectra were recorded on either Bruker Avance 300, 400 or 500 or JEOL 400 YH spectrometers operating at 75.48 MHz, 100.54 MHz and 125.85 MHz, respectively. All ^{1}H and ^{13}C NMR chemical shifts are reported in ppm relative to $SiMe_4$ using the ^{1}H and ^{13}C chemical shifts of the solvent[5] as a standard. ^{1}H NMR data for diamagnetic compounds are reported as follows: chemical shift, multiplicity (s = singlet, d = doublet, t = triplet, q = quartet, p = pentet, br = broad, m = multiplet, app = apparent, obsc = obscured), coupling constants (Hz), integration, assignment. ^{1}H NMR data for paramagnetic compounds are reported as follows: chemical shift, integration, peak width at half height (Hz). ^{13}C NMR data for diamagnetic compounds are reported as follows: chemical shift, number of protons attached to carbon (e.g. CH_2), assignment. ^{19}F NMR spectra were recorded on Bruker Avance 400 or 500 spectrometers operating at 376.19 MHz and 470.96 MHz,

respectively, or JEOL 400YH at 396 MHz and referenced to $CFCl_3$ as an external standard. IR Spectra were collected on a Thermo Fischer Nicolet iS20 FTIR spectrometer neat on KBr plates. High-resolution mass spectra were obtained at Princeton University mass spectrometry facilities using either an Agilent 6230 TOF LC/MS, Agilent 7200 Accurate-Mass Q-TOF GC/MS or 7250 GC/Q-TOF.

Separation of certain borylation product of $(OC)_3Cr(\eta^6$-arene) was performed by preparative chiral SFC performed by Lotus Separations, LLC, with a Mettler SFC supercritical CO_2 analytical chromatography system utilizing Chiralpak (ADH, AS-H or IC) or Chiralcel (OD-H, OJ-H, or OB-H) columns (4.6 mm x 25 cm) obtained from Daicel Chemical Industries, Ltd.

II. Preparation of Cobalt Precatalyst

Preparation of ^{iPr}ACNCCo$_2$(O^{t}Bu)$_4$ (5). The catalyst **5** was prepared by our previously published synthesis.[2] In the glovebox, $K_2Co_2(O^tBu)_6$ (0.34 g, 0.54 mmol) was weighed into a 100 mL round bottom flask equipped with a stir bar and toluene (25 mL) was added. It was put into a cold well and chilled for about 15 minutes. ^{iPr}ACNC(HBr)$_2$ was added in small batches over about 5 minutes with vigorous stirring. The mixture was allowed to warm to room temperature and stir for about 18 hours. The volatiles were removed under vacuum and the solid resuspended in toluene (ca 10 mL) and subsequently filtered through celite. The residue was washed with toluene (3 x 5 mL) and the combined filtrates were evaporated to dryness. The residue was dissolved in 3 mL diethyl ether, filtered through celite, and the celite washed with ether until the eluent was colorless; the ether was subsequently removed under vacuum. The residual solid was triturated with pentane to give 0.108 g (0.184 mmol, 49% yield) of desired ^{iPr}ACNCCo$_2$(O^{t}Bu)$_4$.

^{1}H NMR (400 MHz, benzene-d_6, 23 °C): δ 42.96 (s, 2H), 42.13 (s, 2H), 38.49 (s, 6H), 9.33 (s, 5H), 7.10 (s, 18H), -3.98 (s, 1H), -6.29 (s, 6H), -26.75 (s, 9H), -28.97 (s, 9H).

III. Preparation of Tricarbonyl Chromium η^6-Arene Complexes

General Procedure. A method modified from that published by Larrosa was used.[6] Arene (*ca* 4 mmol, 1 equiv) and $Cr(CO)_6$ (*ca* 1.3 equiv) were added to a 50 mL round bottomed flask connected to an air condenser and the apparatus put under argon on the Schlenk line. Di-n-butyl ether (Sigma, anhydrous) and sieve-dried THF (*ca* 9 to 1 ratio) were added via syringe to give a 0.15 M solution. The colorless mixture was degassed using three freeze pump thaw cycles, opened to an argon bubbler then heated to reflux in a 145 °C oil bath. The reaction was followed by TLC to full completion, usually within 48 hours, during which time the solution became yellow in color. The reaction was then cooled under argon, the volatile components removed by rotary evaporation, and the typically yellow solid purified either by flash silica chromatography or by trituration with hexanes or diethyl ether.

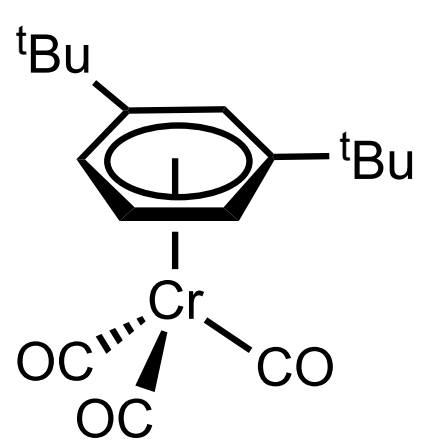

1,3-di(^{t}Bu)phenyl chromium tricarbonyl (4a) was prepared from 1,3-di-*tert*-butylbenzene (0.800 g, 4.2 mmol) and $Cr(CO)_6$ (1.18 g, 5.3 mmol) to yield 0.568 g (1.72 mmol, 41%) of a yellow solid after removing the volatiles. Analytical data was consistent with literature values for 1,3-di(^{t}Bu)phenyl chromium tricarbonyl.[7]

^{1}H NMR (400 MHz, chloroform-*d*, 23 °C): δ 5.67 (s, 1H), 5.52 (d, *J* = 6.6 Hz, 2H), 5.03 (t, *J* = 6.6 Hz, 1H), 1.25 (s, 18H).

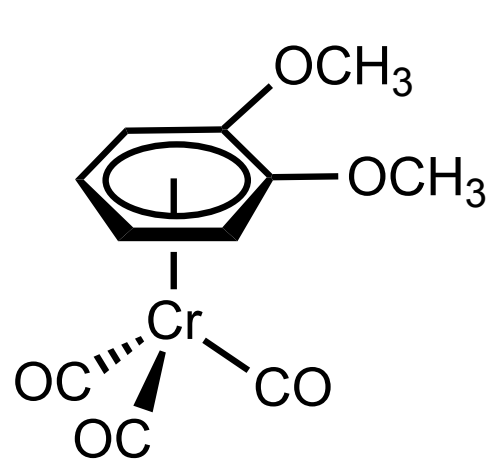

1,2-dimethoxyphenyl chromium tricarbonyl (6a) was prepared from 1,2-dimethoxybenzene (0.41 g, 3.0 mmol) and $Cr(CO)_6$ (0.66 g, 3.0 mmol) to yield 0.18 g (0.66 mmol, 22%) of yellow solid after removing the volatiles. Analytical data was consistent with literature values for the desired material.[7]

^{1}H NMR (500 MHz, acetone-d_6, 23 °C): δ 5.72 (bs, 2H), 5.34 (bs, 2H), 3.80 (s, 6H).

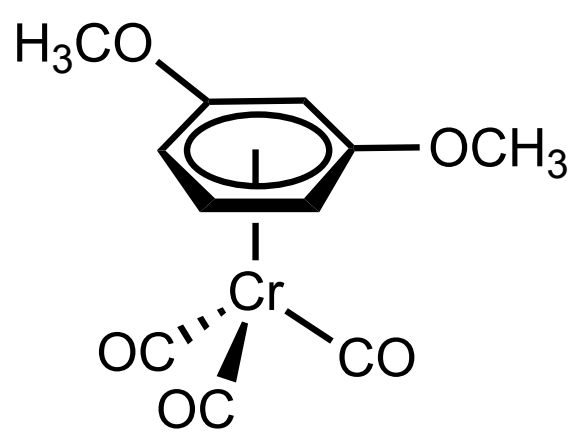

1,3-di(methoxy)phenyl chromium tricarbonyl (7a) was prepared from 1,3-dimethoxybenzene (0.55 g, 4.0 mmol) and Cr(CO)6 (1.07 g, 4.9 mmol) for 37 h. The yellow solid resulting from rotary evaporation was dissolved in ethyl acetate and filtered through celite. Evaporation yielded a bright yellow solid (0.74 g, 2.8 mmol, 71%). Analytical data was consistent with literature values for the desired material.[7]

^{1}H NMR (400 MHz, acetone-d_6, 23 °C): δ 5.91 (t, J = 6.8 Hz, 1H), 5.50 (t, J = 2.1 Hz, 1H), 5.17 (dd, J = 6.8, 2.1 Hz, 2H), 3.81 (s, 6H).

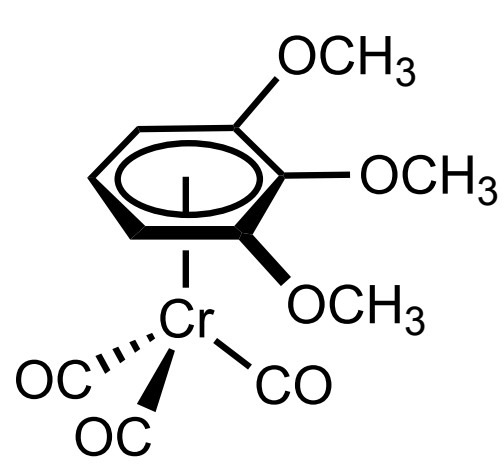

1,2,3-trimethoxybenzene chromium tricarbonyl (8a) was prepared by the standard method. 1,2,3-trimethoxybenzene (0.41 g, 2.4 mmol) and $Cr(CO)_6$ (0.81 g, 3.7 mmol) were refluxed for 20 hours. The reaction mixture was filtered through a short plug of SiO_2 (ethyl acetate) then the volatiles were removed via rotary evaporation. NMR showed approximately 10% starting material

remained, which was removed by vacuum. The product was isolated as a yellow powder after evaporation of the CH_2Cl_2 (0.628 g, 2.1 mmol, 84%). Analytical data was consistent with literature values for the desired material.[7]

^{1}H NMR (400 MHz, acetone-*d*$_6$, 23 °C): δ 5.75 (t, *J* = 6.8 Hz, 1H), 5.24 (d, *J* = 6.8 Hz, 2H), 3.88 (d, *J* = 7.1 Hz, 6H), 3.82 (s, 3H).

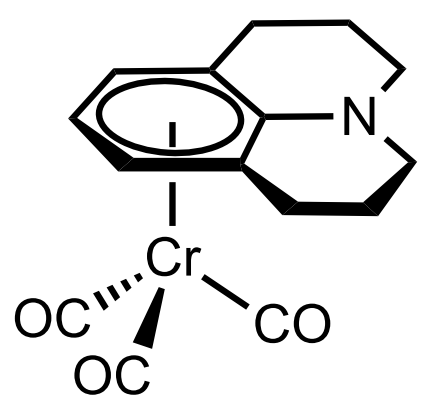

2,3,6,7-Tetrahydro-*1H,5H*-pyrido[3,2,1-*ij*]quinoline chromium tricarbonyl (9a) (julolidine chromium tricarbonyl) was prepared from julolidine (0.625 g, 3.6 mmol) and $Cr(CO)_6$ (1.00 g, 4.5 mmol) to yield 0.765 g (2.5 mmol, 69% yield) of bright yellow solid after removing the volatiles and chromatography (SiO_2, diethyl ether). Analytical data was consistent with literature values for the desired material.[7]

^{1}H NMR (500 MHz, chloroform-*d*, 23 °C): δ 6.88 (d, *J* = 7 Hz, 2H), 6.59 (t, *J* = 7 Hz, 1H), 3.22 (m, 4H), 2.85 (m, 4H), 2.07 (m, 4H).

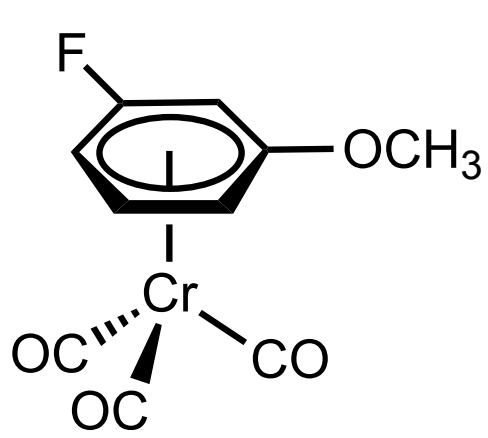

3-Fluoroanisole chromium tricarbonyl (10a) was prepared by the standard method. 3-fluoroanisole (0.448 g, 3.5 mmol) and $Cr(CO)_6$ (0.97 g, 4.5 mmol) were refluxed for 48 hours. Crystallization of the residue with hexane gave the desired product (0.337 g, 1.3 mmol, 37%) as a yellow powder.

^{1}H NMR (400 MHz, acetone-*d*$_6$, 23 °C): δ 5.99 (td, *J* = 6.8, 3.7 Hz, 1H), 5.86 (dt, *J* = 6.5, 2.0 Hz, 1H), 5.47 (ddd, *J* = 6.7, 4.4, 2.0 Hz, 1H), 5.24 (dt, *J* = 6.8, 2.0 Hz, 1H).

^{13}C NMR (75 MHz, acetone-*d*$_6$, 23 °C): δ 234.07, 94.56, 94.45, 90.72, 82.72, 76.85, 76.55, 76.27, 71.30, 71.00, 56.93.

^{19}F NMR (400 MHz, acetone-*d*$_6$, 23 °C): δ -135.23.

IR (KBr, neat, cm^{-1}) 3106, 1956, 1873, 1541, 1524, 1468, 1294, 1164, 1140, 1033, 992, 824, 671, 655, 631.

HRMS (ESI+) *m/z* calculated for [$C_{10}H_8CrFO_4^+$] ([M+H]$^+$) 262.9806, found *m/z* 262.9801.

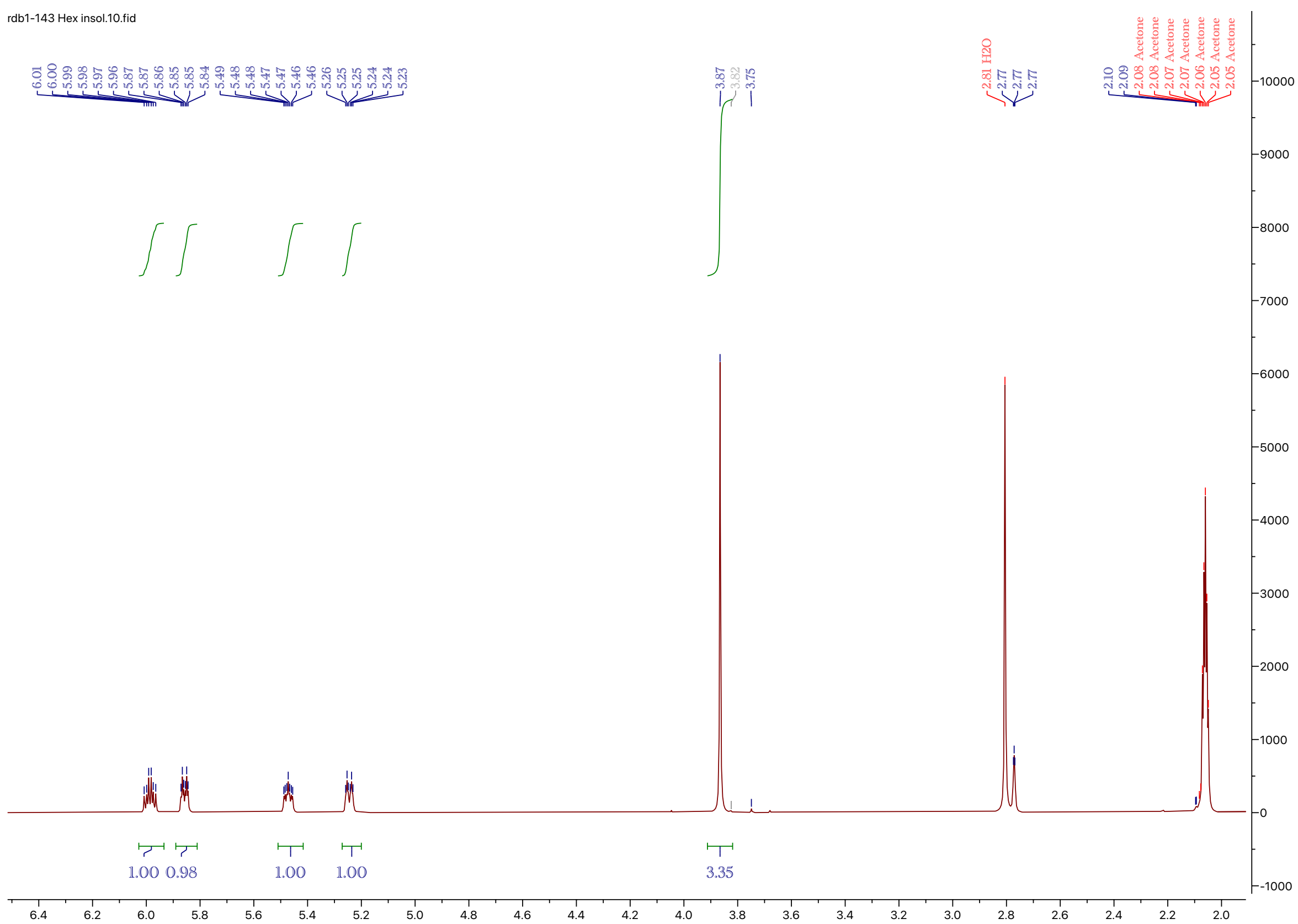

***Figure S1**.* ^{1}H NMR spectrum (400 MHz, acetone-*d*$_6$, 23 °C) of 3-Fluoroanisole chromium tricarbonyl (**10a**).

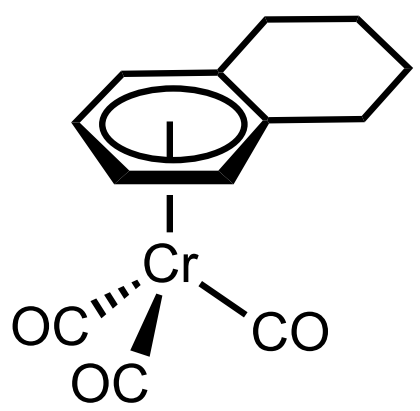

1,2,3,4-Tetrahydronaphthalene chromium tricarbonyl (11a) was prepared by the standard method. Tetralin (0.45 mL, 3.5 mmol) and $Cr(CO)_6$ (0.89 g , 4.1 mmol) were refluxed for 16 hours. The resulting powder was placed under vacuum for several hours to remove residual tetralin resulting in a bright yellow powder (0.804 g, 3.0 mmol, 86%). Analytical data was consistent with literature values for the desired material.[7]

^{1}H NMR (400 MHz, acetone-*d*$_6$, 23 °C): δ 5.57 – 5.48 (m, 4H), 2.79 – 2.54 (m, 4H), 1.76 (qt, *J* = 6.1, 2.7 Hz, 4H).

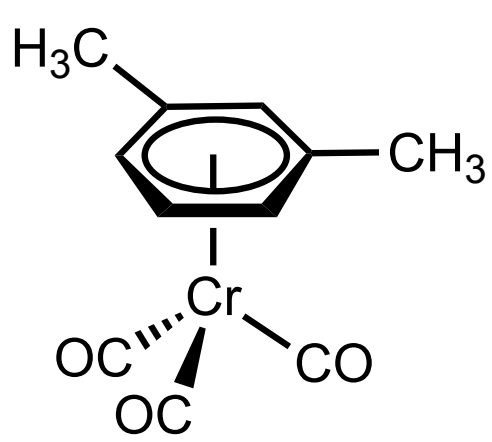

1,3-dimethylbenzene chromium tricarbonyl (12a) was prepared by a modified procedure. 1,3-dimethylbenzene (0.9 mL, 4.7 mmol) and $Cr(CO)_6$ (1.36 g , 6.1 mmol) were refluxed for 24 hours at which time additional 1,3-dimethylbenzene (0.3 mL, 1.6 mmol) was added. Reflux for a further 24 hours yielded the desired product (1.26 g, 4.9 mmol, 81%) of yellow powder. Analytical data was consistent with literature values for the desired material.[7]

^{1}H NMR (400 MHz, Chloroform-*d*, 23 °C): δ 5.45 (t, *J* = 6.3 Hz, 1H), 5.04 – 4.96 (m, 2H), 2.20 (s, 6H).

^{13}C NMR (101 MHz, Chloroform-*d*, 23 °C): δ 233.77, 110.57, 95.38, 93.98, 90.30, 20.91.

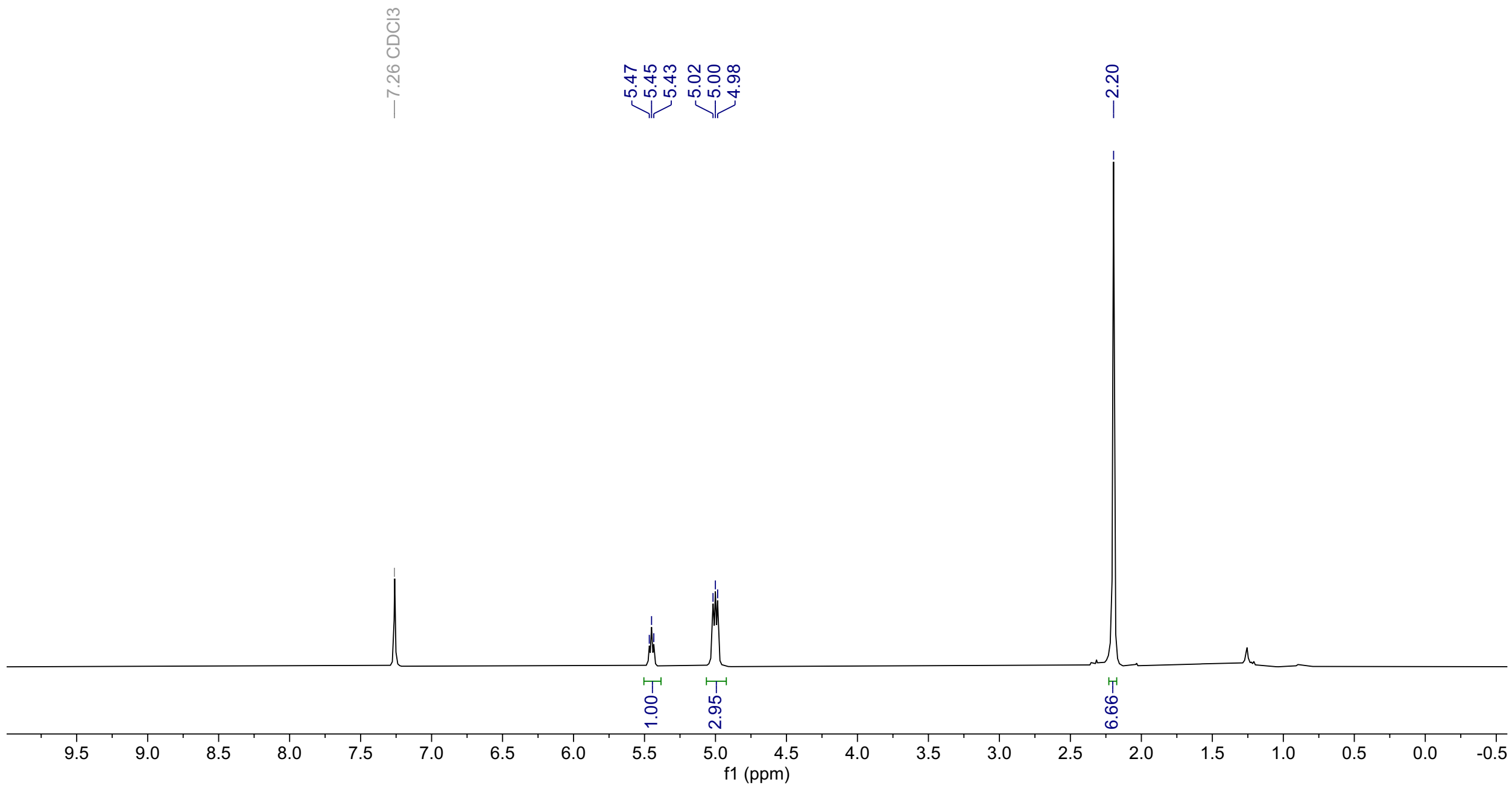

Figure S2. ^{1}H NMR spectrum (400 MHz, Chloroform-*d*, 23 °C) of 1,3-dimethylbenzene chromium tricarbonyl (**12a**).

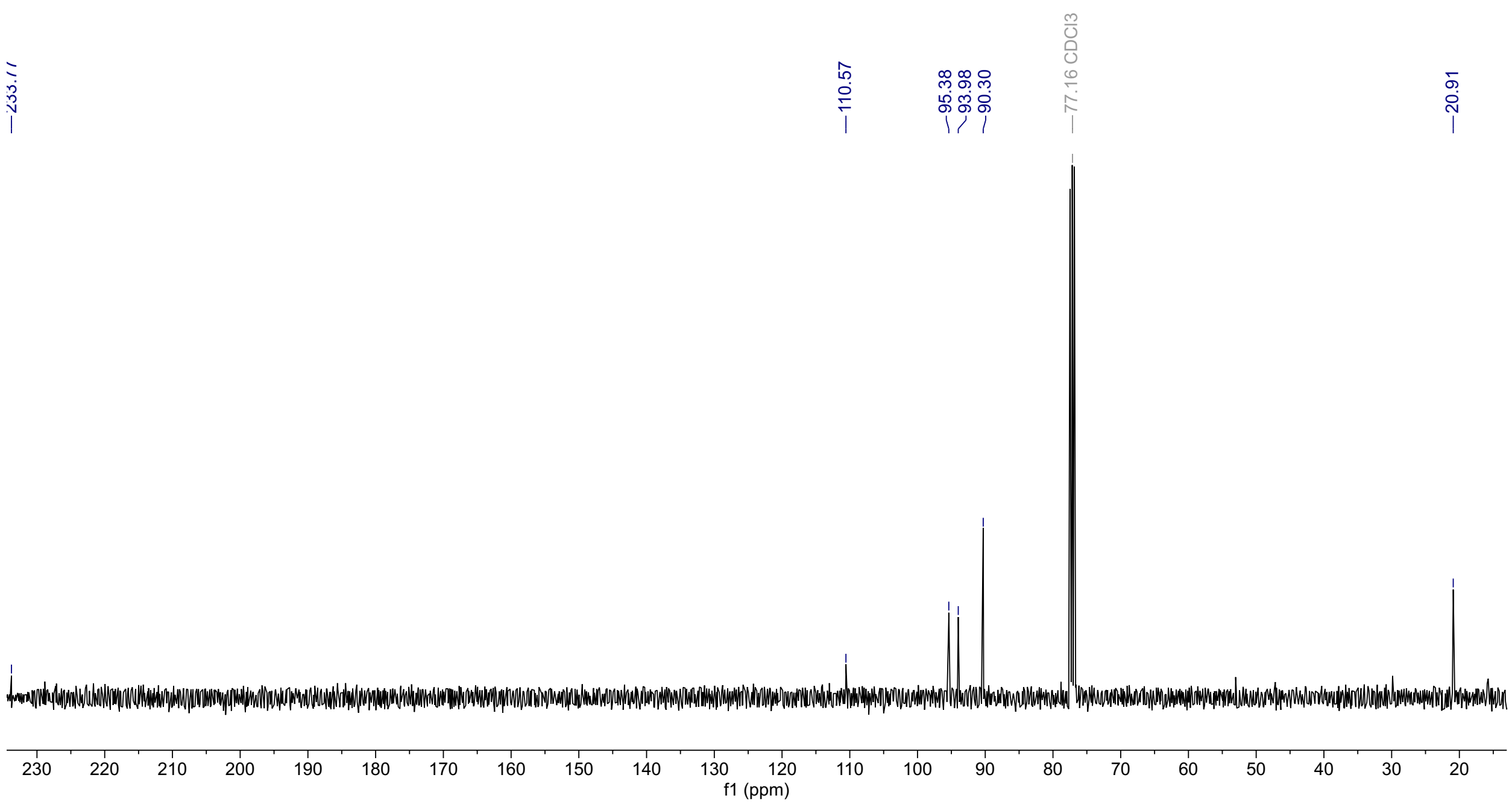

Figure S3. ^{13}C NMR spectrum (101 MHz, Chloroform-*d*, 23 °C) of 1,3-dimethylbenzene chromium tricarbonyl (**12a**).

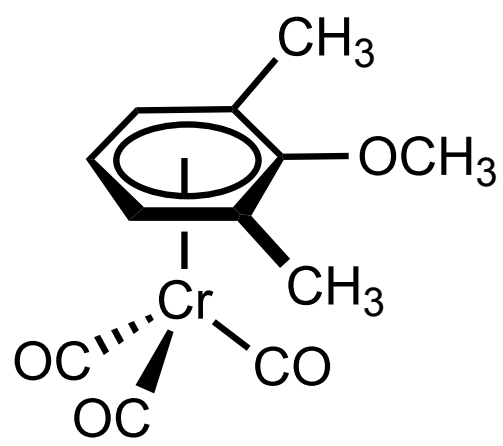

2,6-dimethylanisole chromium tricarbonyl (13a) was prepared by the standard method. 2,6-dimethylanisole (0.514 g, 3.7 mmol) and $Cr(CO)_6$ (1.0 g, 4.4 mmol) were refluxed for 21 hours. Crystallization of the residue with hexane gave the desired product (0.710 g, 2.6 mmol, 69%) as a yellow powder. Analytical data was consistent with literature values for the desired material.[8]

^{1}H NMR (300 MHz, acetone-*d*$_6$, 23 °C): δ 5.54 – 5.37 (m, 3H), 3.78 (s, 3H), 2.28 (s, 6H).

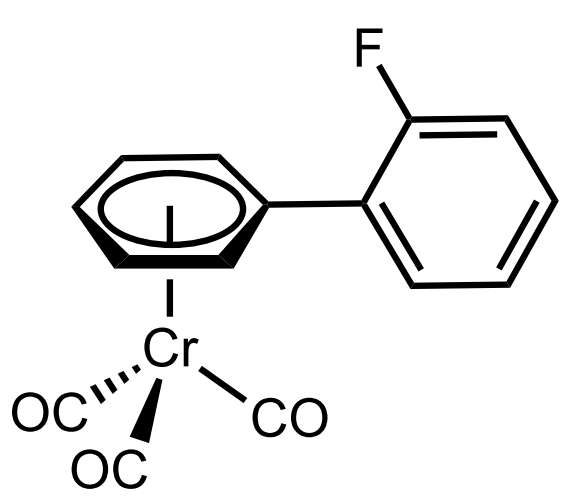

1-(2-Fluorophenyl)benzene chromium tricarbonyl (14a) was prepared by the standard method. 2-fluorobiphenyl (0.54 g, 3.1 mmol) and $Cr(CO)_6$ (0.94 g , 4.2 mmol) were refluxed for 48 hours. Trituration of the solid with hexane gave the desired product (0.258 g, 0.8 mmol, 26%) of yellow powder after trituration with hexane.

^{1}H NMR (300 MHz, chloroform-*d*, 23 °C): δ 7.55-7.10 (m, 5H), 5.71 (d, 2H), 5.42 (t, 2H).

^{13}C NMR (101 MHz, chloroform-*d*, 23 °C): δ 232.50, 161.24, 158.73, 130.78, 130.59 (d),124.70, 116.28 (d), 104.60, 94.70, 92.27, 91.47.

^{19}F NMR (282 MHz, chloroform-*d*, 23 °C): δ -115.44.

IR (neat, cm^{-1}) 1966, 1887, 1468, 1457, 1252, 1205, 1150, 1107, 819, 763.

HRMS (ESI+) *m/z* calculated for [$C_{15}H_{10}CrFO_3^+$] ($[M+H]^+$) 309.0014, found *m/z* 309.0013.

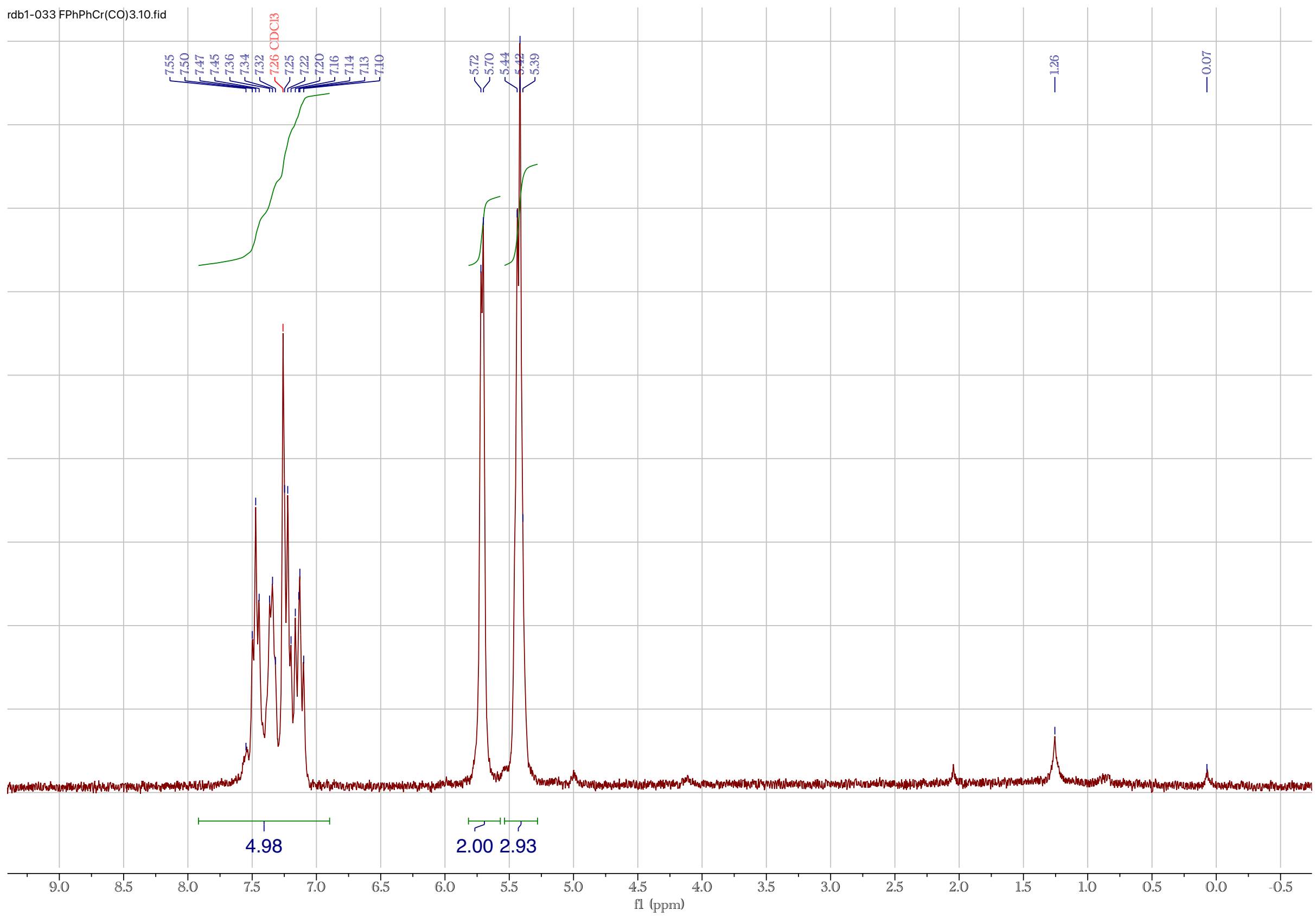

Figure S4. ^{1}H NMR spectrum (300 MHz, chloroform-*d*, 23 °C) of (2-fluorophenyl)benzene chromium tricarbonyl complex **14a**.

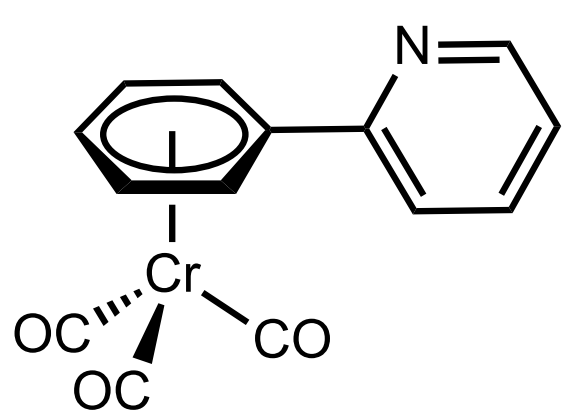

(2-pyridyl)benzene chromium tricarbonyl was prepared by the standard method. 2-Phenylpyridine (0.60 g, 4.9 mmol) and $Cr(CO)_6$ (1.15 g, 5.2 mmol) were refluxed for 48 hours. Trituration of the solid with hexane gave a gold-colored powder (0.90 g) which was about 90% pure. Chromatography (SiO2, hexane/EtOAc, 1/1) gave the desired material (0.138 g, 0.47 mmol, 10% yield) as an orange solid. Analytical data was consistent with literature values for the desired material.[9]

^{1}H NMR (400 MHz, chloroform-*d*, 23 °C): δ 8.75 – 8.51 (m, 1H), 7.75 (t, *J* = 7.8 Hz, 1H), 7.56 (d, *J* = 8.0 Hz, 1H), 7.26 (1H), 6.18 (d, *J* = 6.4 Hz, 2H), 5.51 (t, *J* = 6.3 Hz, 2H), 5.43 (t, *J* = 6.1 Hz, 1H).

^{13}C NMR (101 MHz, Chloroform-*d*, 23 °C): δ 232.63, 149.70, 137.05, 123.63, 120.15, 92.49, 92.25, 91.92, 77.48, 77.16, 76.84.

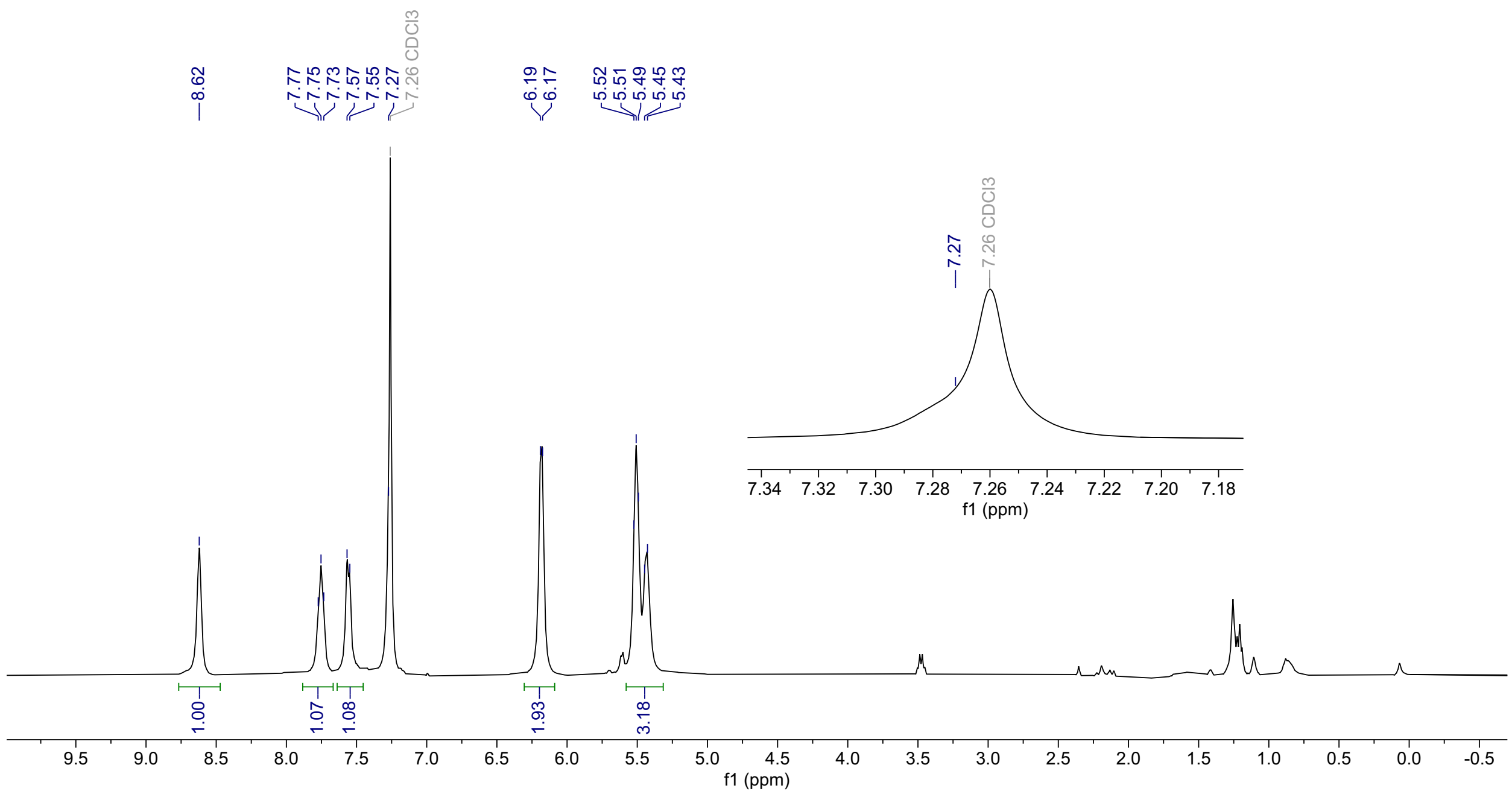

Figure S5. ^{1}H NMR spectrum (400 MHz, chloroform-*d*, 23 °C) of the (2-pyridyl)benzene chromium tricarbonyl complex.

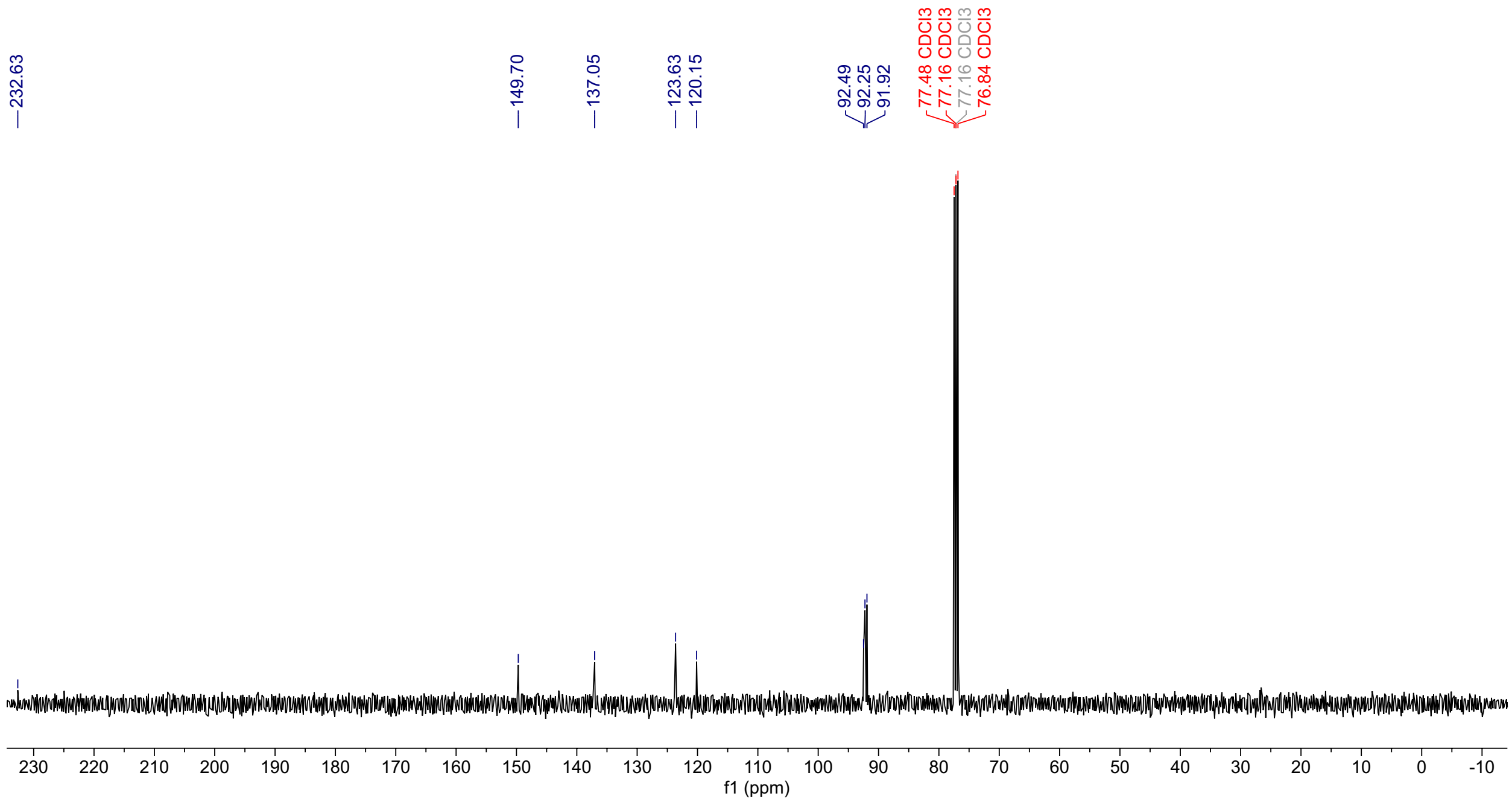

Figure S6. ^{13}C NMR spectrum (101 MHz, chloroform-*d*, 23 °C) of the (2-pyridyl)benzene chromium tricarbonyl complex.

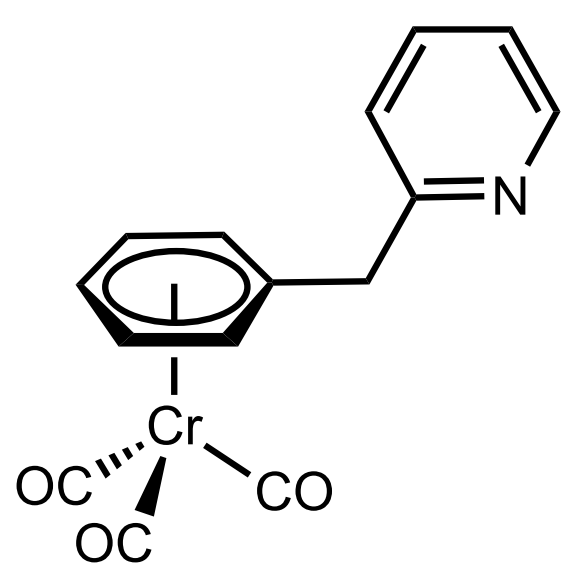

(2-Pyridylmethyl)benzene chromium tricarbonyl was prepared by the standard method. 2-benzylpyridine (0.32 mL, 1.9 mmol) and $Cr(CO)_6$ (0.55 g, 2.5 mmol) were refluxed for 16 hours. Substantial decomposition took place to give metallic chromium. The mixture was filtered through celite then the volatile materials removed by rotary evaporation to give a yellow oil, which was crystallized from boiling hexane to yield a yellow solid (0.138 g, 0.44 mmol, 24%). Analytical data was consistent with literature values for the desired material.[10]

^{1}H NMR (400 MHz, acetone-d_6, 23 °C): δ 8.51 (dt, *J* = 4.7, 1.5 Hz, 1H), 7.73 (td, *J* = 7.7, 1.9 Hz, 1H), 7.41 (d, *J* = 7.8 Hz, 1H), 7.23 (ddd, *J* = 7.7, 4.8, 1.2 Hz, 1H), 5.65 (m, 4H), 5.50 (m, 1H), 3.86 (s, 2H).

^{13}C NMR (101 MHz, acetone-d_6, 23 °C): δ 233.81, 149.45, 136.76, 123.02, 121.96, 94.84, 94.79, 92.26, 42.97.

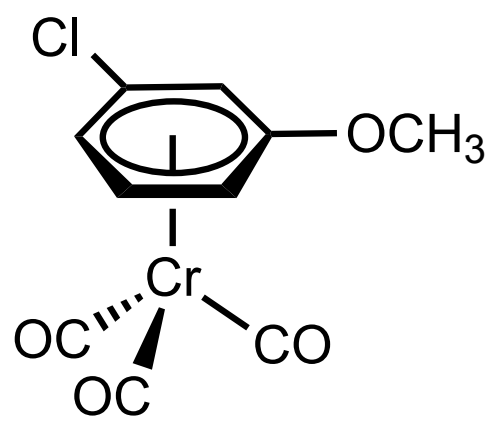

3-chloroanisole chromium tricarbonyl was prepared by the standard method. 3-chloroanisole (0.50 g, 3.5 mmol) and $Cr(CO)_6$ (0.90 g , 4.1 mmol) were refluxed for 19 hours. Reaction showed considerable decomposition. The volatiles were removed via rotary evaporation and the solids suspended in 1/1 Et_2O/CH_2Cl_2 and filtered through a plug of SiO_2 using the same to elute, giving an orange solid. Crystallization of the residue with hexanes gave the desired product (0.115 g, 0.44 mmol, 12%) of yellow powder. Analytical data was consistent with literature values for the desired material.[7]

^{1}H NMR (500 MHz, chloroform-*d*, 23 °C): δ 5.55 (td, *J* = 6.5, 3.2 Hz, 1H), 5.35 (t, *J* = 1.8 Hz, 1H), 5.09 (dd, *J* = 6.4, 1.7 Hz, 1H), 4.91 (dd, *J* = 6.7, 2.1 Hz, 1H), 3.75 (s, 3H).

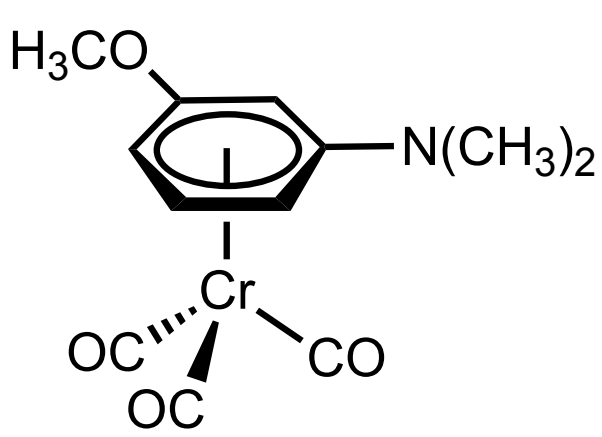

3-methoxy-N,N-dimethylaniline chromium tricarbonyl (16a) was prepared by the standard method. 3-methoxy-*N*,*N*-dimethylaniline (0.51 mL, 3.4 mmol) and $Cr(CO)_6$ (0.75 g, 3.4 mmol) were refluxed for 47 hours. After filtration, the volatiles were removed via rotary evaporation and the solids were triturated with hot hexanes, then dissolved in a minimum amount of CH_2Cl_2 and filtered through a short plug of SiO_2 using CH_2Cl_2 as eluent. The product was isolated as a yellow

powder after evaporation of the CH_2Cl_2 (0.447 g, 1.56 mmol, 40%). Analytical data was consistent with literature values for the desired material.[7]

^{1}H NMR (400 MHz, acetone-d_6, 23 °C): δ 5.83 (t, *J* = 6.9 Hz, 1H), 5.14 (t, *J* = 2.2 Hz, 1H), 5.08 (dd, *J* = 6.7, 2.1 Hz, 1H), 4.84 (dd, *J* = 7.0, 2.3 Hz, 1H), 4.06 (q, *J* = 7.1 Hz, 1H), 3.78 (s, 3H), 2.97 (s, 6H).

IV. General Catalytic Procedures and Products

General Procedure: Borylation of Arene-Chromium Tricarbonyl Complexes. A method modified from that published by Shimozono *et al.* was used.[11] In a typical experiment, in a nitrogen-filled glovebox, to a 5 dram vial was added (^{iPr}ACNC)Co_2(O^{t}Bu)$_4$ (0.005 mmol, 0.05 equiv.), 0.025 g of B_2Pin_2 (0.10 mmol, 1.1-2 equiv.), arene substrate (0.10 mmol, 1.0 equiv.), 1 mL of THF, and a stir bar. Alternatively, stock solutions of B_2Pin_2, (^{iPr}ACNC)Co_2(O^{t}Bu)$_4$ and 1,3,5-trimethoxybenzene were prepared in THF and appropriate amounts were added to the substrate in THF. The mixture was allowed to stir for 1-24 hours at ambient temperature. The reaction was removed from the glove box, 5.0 mg of 1,3,5-trimethoxybenzene added, diluted with diethyl ether or chloroform and filtered through a thin pad of silica gel under air. The resulting mixture was concentrated and then analyzed by ^{1}H and ^{13}C NMR spectroscopies. Most of the products were previously reported and were spectroscopically assigned as the desired products (without isolation) by comparison to reported spectra using 1,3,5-trimethoxybenzene as the internal integration standard.

Note: Upon standing in NMR solvents, a precipitate formed in nearly all cases and resonances consistent with chromium-free arene were observed. This is consistent with previous observations that chromium tricarbonyl arene complexes slowly undergo decomposition when in solution and especially when light is present.

Control reaction without added catalyst. 1,3-di(^{t}Bu)phenyl chromium tricarbonyl (**4a**, 0.20 g 0.073 mmol) and B_2Pin_2 (0.018 g, 0.071 mmol) were added to a vial containing a stir bar. The vial was capped, the solution stirred and allowed to stir in the glovebox. After 48 hours, 1,3,5-trimethoxybenzene (5.0 mg) was added and then an aliquot filtered through SiO_2 (chloroform-*d*). ^{1}H NMR indicated that no reaction had occurred.

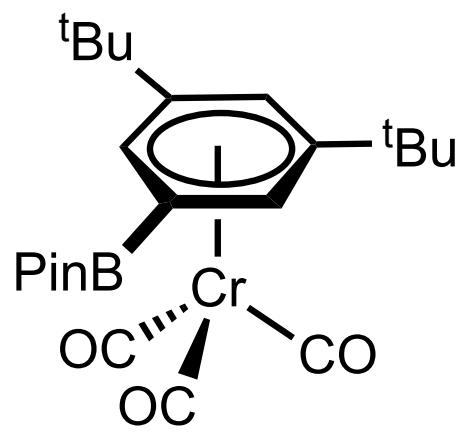

2-(3,5-Di-*tert*-butylphenyl)-4,4,5,5-tetramethyl-1,3,2-dioxaborolane chromium tricarbonyl (4b) was prepared by adding 1,3-di(^{t}Bu)phenyl chromium tricarbonyl (**4a**, 0.20 g, 0.066 mmol), B_2Pin_2 (0.017 g, 6.7 mmol), **5** (0.003 g, 0.00030 mmol), and 1,3,5-trimethoxybenzene (0.005 g) to a vial containing a stir bar. The vial was capped, stirred and the solution turned light brown. After 1 hour, quantitative NMR data showed an 81% yield of **4b** with less than 2% of **4a** remaining after evaporation of the volatile material and chromatography (SiO_2, $CHCl_3$). Analytical data was consistent with literature values for the desired material.[12]

^{1}H NMR (400 MHz, chloroform-*d*, 23 °C): δ 5.92 (bs, 3H), 1.32 (s, 12H), 1.29 (s, 18H).

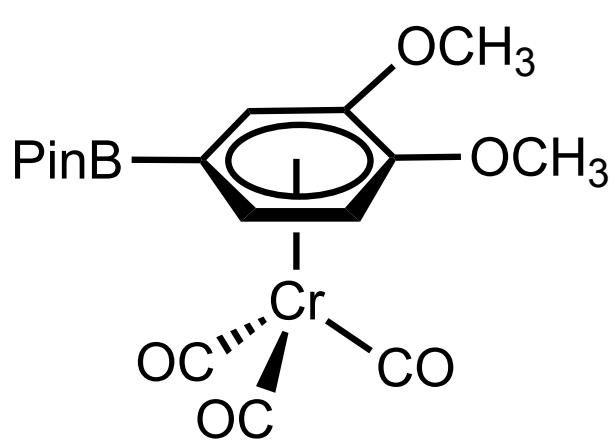

2-(3,4-Dimethoxyphenyl)-4,4,5,5-tetramethyl-1,3,2-dioxaborolane chromium tricarbonyl (6b) was prepared by the addition of 1,2-dimethoxyphenyl chromium tricarbonyl (**6a**, 0.018 g, 0.066 mmol) in THF (0.4 mL) to stock solutions of B_2Pin_2 (0.2 mL, 0.33 M, 0.066 mmol) and **5** (0.1 mL, 0.048 M, 0.0048 mmol) along with 1,3,5-trimethoxybenzene (0.005 g). After two hours the reaction was removed from the glovebox, the volatiles evaporated and the residue filtered through SiO_2 ($CHCl_3$). Analytical data was consistent with literature values for the desired material and quantitative ^{1}H NMR indicated a 58% yield of **6b**.[12]

^{1}H NMR (300 MHz, chloroform-*d*, 23 °C): δ 5.62 (bs, 1H), 5.50 (d, *J* = 6.6 Hz, 1H), 5.19 (dd, *J* = 6.6, 0.4 Hz, 1H), 3.84 (s, 6H), 1.39 (s, 12H).

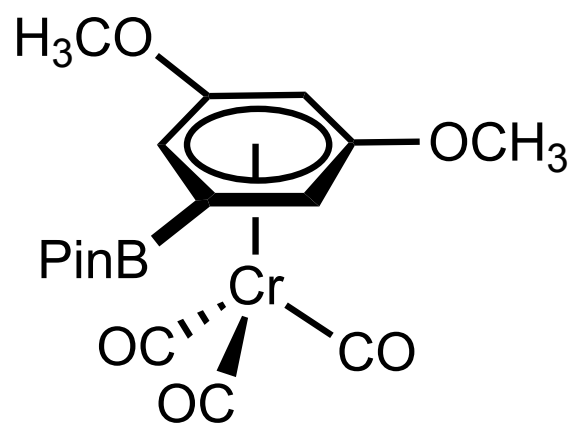

2-(3,5-Dimethoxyphenyl)-4,4,5,5-tetramethyl-1,3,2-dioxaborolane chromium tricarbonyl (**7b**) was prepared by the addition of 1,3-dimethoxyphenyl chromium tricarbonyl (**7a**, 0.029 g, 0.10 mmol) to B_2Pin_2 (0.029 g, 0.11 mmol), **5** (0.20 mL, 0.022 M in THF, 0.0044 mmol) and THF (0.8 mL). After 4.5 hours, the reaction was opened to air and 1,3,5-trimethoxybenzene (5.0 mg) was added. The volatile materials were evaporated under a stream of nitrogen and the residue chromatographed on SiO_2. Analytical data was consistent with literature values for the desired material and quantitative ^{1}H NMR showed a 97% yield of **7b**.[12]

^{1}H NMR (300 MHz, acetone-d_6, 23 °C): δ 5.60 (t, J = 2 Hz, 1H), 5.08 (d, J = 2 Hz, 2H), 3.82 (s, 6H), 1.31 (s, 12H).

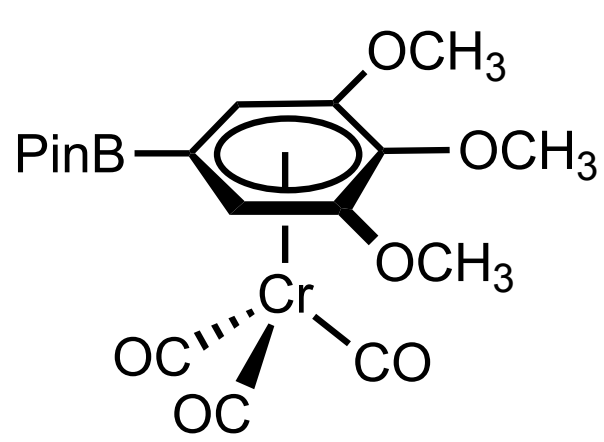

4,4,5,5-Tetramethyl-2-(3,4,5-trimethoxyphenyl)-1,3,2-dioxaborolane chromium tricarbonyl (8b) was prepared by the general procedure using **8a** (0.030 g, 0.098 mmol), **5** (2.3 mg, 0.05 equiv.), B_2Pin_2 (0.026 g, 0.11 mmol) and 1 mL THF. The reaction was capped and stirred for 5 hours; the color turned brown after about 50 minutes. After removing from the box 1,3,5-trimethoxybenzene was added and the volatile materials removed under a stream of dry nitrogen gas, and chromatographed (SiO_2, diethyl ether). Analytical data was consistent with literature values for the desired material and quantitative ^{1}H NMR showed an 85% yield of **8b**.[12]

^{1}H NMR (400 MHz, acetone-d_6, 23 °C): δ 5.14 (s, 2H), 3.89 (s, 6H), 3.86 (s, 3H), 1.37 (s, 12H).

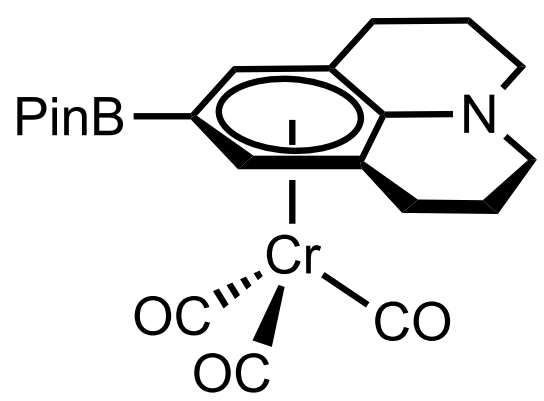

9-(4,4,5,5-Tetramethyl-1,3,2-dioxaborolan-2-yl)-2,3,6,7-tetrahydro-1*H*,5*H*-pyrido[3,2,1-*ij*]quinoline chromium tricarbonyl (**9b**) was prepared by the general procedure using 9a (0.020 g, 0.067 mmol), 5 (0.05 equiv), B_2Pin_2 (1.1 equiv), 1,3,5-trimethoxybenzene (5.0 mg), and 0.8 mL THF. The color turned brown. The reaction was capped and stirred for 18 hours. Analytical data was consistent with literature values for the desired material and ^{1}H NMR showed an 86% yield of **9b**.[12]

^{1}H NMR (300 MHz, chloroform-*d*, 23 °C): δ 5.73 (s, 2H), 3.12-3.25 (m, 2H), 2.52-2.63 (m, 2H), 2.03-2.18 (m, 2H), 1.83-1.98 (m, 2H), 1.30 (s, 12H).

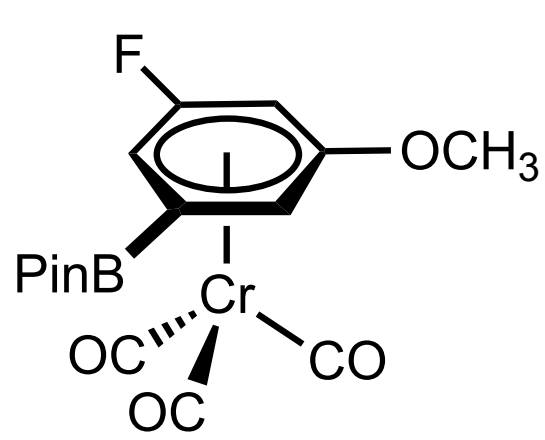

2-(3-fluoro-5-methoxyphenyl)-4,4,5,5-tetramethyl-1,3,2-dioxaborolane chromium tricarbonyl (**10b**) was prepared by the addition of 3-fluoroanisole chromium tricarbonyl (**10a**, 23 mg, 0.088 mmol), B_2Pin_2 (25 mg, 0.098 mmol), **5** (0.35 mg, 0.005 mmol) and THF (1 mL) to a vial containing a stir bar. The vessel was capped and allowed to stir. After 1 hour the reaction was removed from the glovebox, filtered through SiO_2 ($CHCl_3$), and the volatiles were removed by rotary evaporation. Analytical data was consistent with literature values for the desired material and ^{1}H NMR showed an 86% yield of **10b**.

^{1}H NMR (500 MHz, chloroform-*d*, 23 °C): δ 5.71 – 4.56 (m, 4H), 3.76 (s, 3H), 1.26 (s, 12H).

^{13}C NMR (126 MHz, chloroform-*d*, 23 °C): δ 232.24, 91.47, 83.64, 74.09, 69.21 (d, *J* = 22.0 Hz), 56.17 (d, *J* = 10.7 Hz), 25.17.

^{19}F NMR (376 MHz, chloroform-*d*, 23 °C): δ -133.92.

^{19}F NMR (282 MHz, acetone-d_6, 23 °C): δ -133.98.

HRMS (ESI+) *m/z* calculated for [$C_{16}H_{19}BCrFO_6^{+}$] ($[M+H]^{+}$) 389.0658, found *m/z* 389.0659.

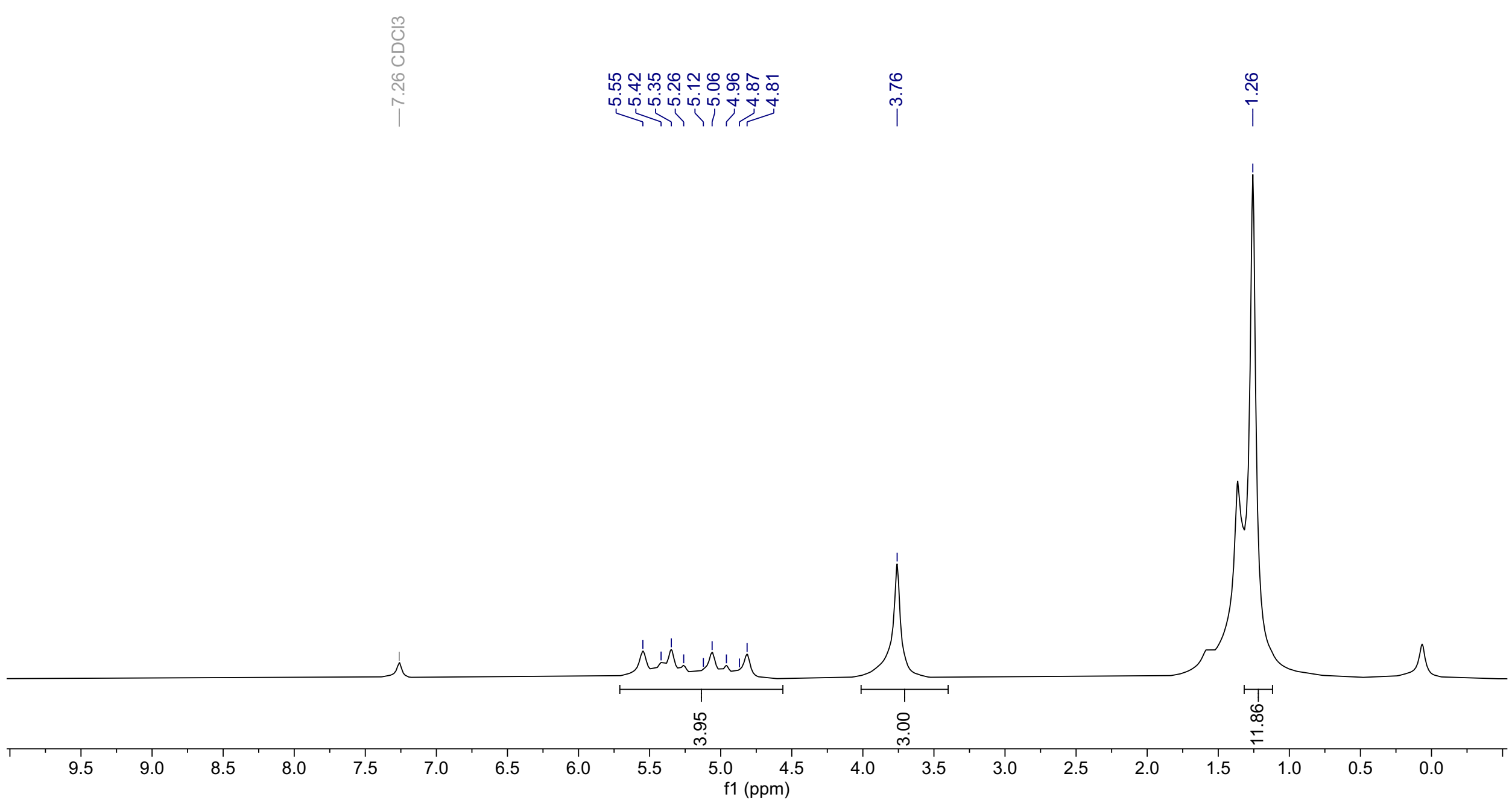

Figure S7. ^{1}H NMR spectrum (500 MHz, chloroform-*d*, 23 °C) of 2-(3-fluoro-5-methoxyphenyl)-4,4,5,5-tetramethyl-1,3,2-dioxaborolane chromium tricarbonyl (**10b**).

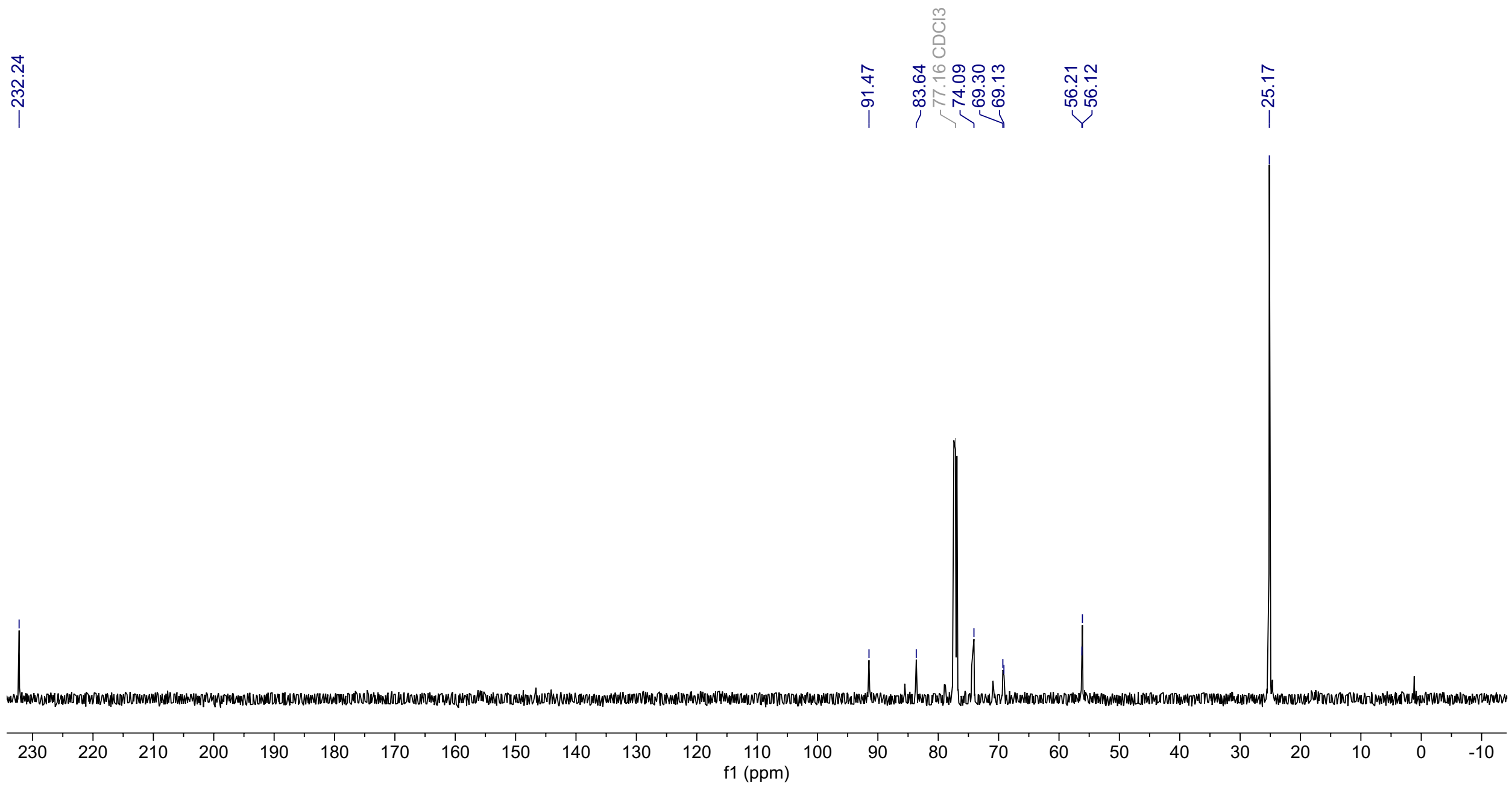

Figure S8. ^{13}C NMR spectrum (126 MHz, chloroform-*d*, 23 °C) of 2-(3-fluoro-5-methoxyphenyl)-4,4,5,5-tetramethyl-1,3,2-dioxaborolane chromium tricarbonyl (**10b**).

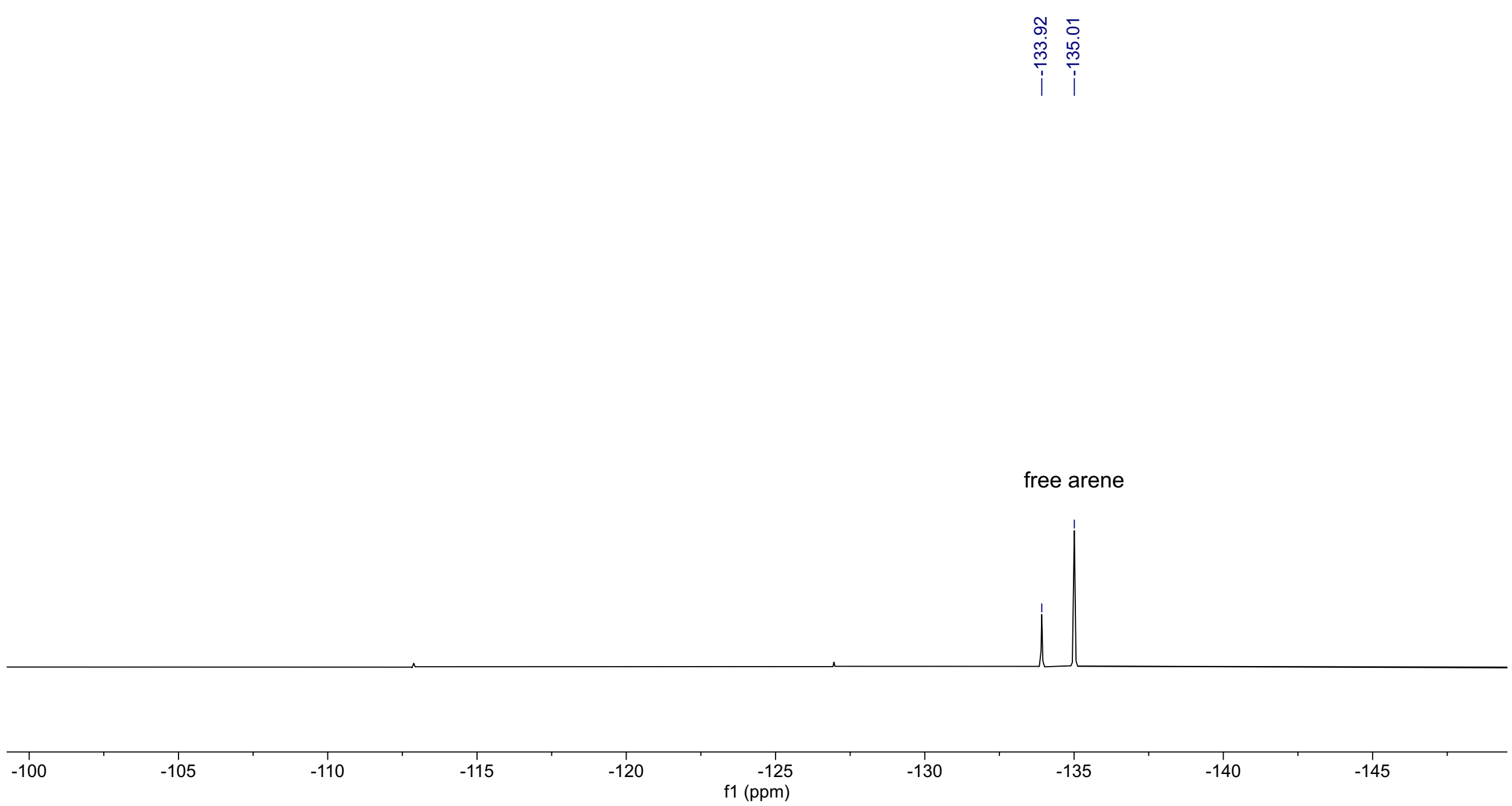

Figure S9. $^{19}F\{^{1}H\}$ NMR spectrum (376 MHz, chloroform-*d*, 23 °C) of 2-(3-fluoro-5-methoxyphenyl)-4,4,5,5-tetramethyl-1,3,2-dioxaborolane chromium tricarbonyl (**10b**).

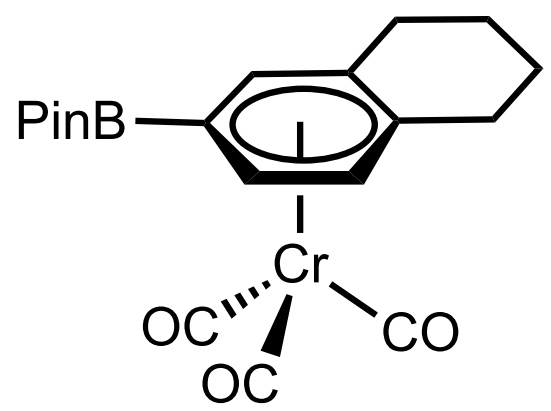

4,4,5,5-Tetramethyl-2-(5,6,7,8-tetrahydronaphthalen-2-yl)-1,3,2-dioxaborolane chromium tricarbonyl (**11b**) was prepared by adding tetralin chromium tricarbonyl (**11a**, 0.023 g, 0.085 mmol) in 0.4 mL THF and stock solutions of B_2Pin_2 (0.2 mL, 0.50 M THF), **5** (0.1 mL, 0.05 M), and 1,3,5-trimethoxybenzene (0.2 mL, 0.157 M) to a vial containing a stir bar. The vial was capped, stirred and the solution turned brown. After one hour, an aliquot was removed, filtered through SiO_2 (diethyl ether) and the volatiles removed under a flow of dry nitrogen. Analytical data was consistent with literature values for the desired material and ^{1}H NMR showed a 93% yield of **11b**.[12]

^{1}H NMR (400 MHz, chloroform-*d*, 23 °C): δ 5.63 (s, 1H), 5.56 (d, J = 5.6 Hz, 1H), 5.10 (d, J = 5.6 Hz, 1H), 2.50-2.80 (m, 4H),1.62-1.88 (m, 4H), 1.30 (s, 12H).

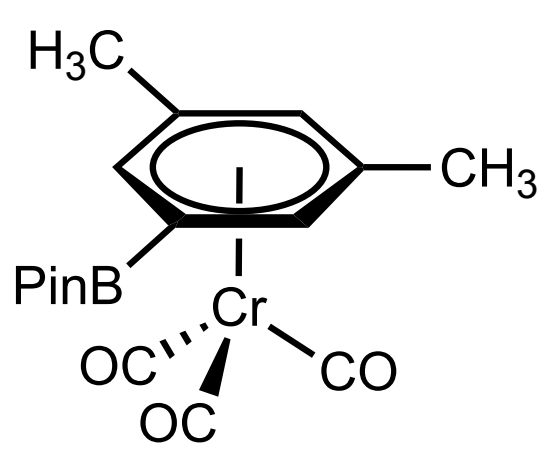

2-(3,5-dimethylphenyl)-4,4,5,5-Tetramethyl-1,3,2-dioxaborolane chromium tricarbonyl (12b) was prepared by adding *m*-xylene chromium tricarbonyl (**12a**, 0.023 g, 0.085 mmol) in 0.4 mL THF and stock solutions of B_2Pin_2 (0.2 mL, 0.50 M in THF), **5** (0.1 mL, 0.05 M), and 1,3,5-trimethoxybenzene (0.2 mL, 0.157 M) to a vial containing a stir bar. The vial was capped, stirred and the solution turned brown. After one hour, an aliquot was removed, filtered through SiO_2 (diethyl ether) and the volatiles removed under a flow of dry nitrogen. Analytical data was consistent with literature values for the desired material and also indicated the presence of the product resulting from additional benzylic borylation in an approximate 2:1 ratio. Detection of the

product from additional benzylic borylation by HRMS (ESI+) was successful (*m/z* calculated for [$C_{23}H_{33}B_2CrO_7^+$] ($[M+H]^+$) 495.1812, found *m/z* 495.1818), but attempts to purify the benzylic borylation product by preparative TLC or chiral SFC were unsuccessful, due to competing decomposition in solution under ambient conditions.

^{1}H NMR (400 MHz, benzene-d_6, 23 °C): δ 5.30 (s, 2H), 4.45 (s, 1H), 1.61 (s, 6H), 1.13 (s, 12H).

HRMS (ESI+) *m/z* calculated for [$C_{17}H_{22}BCrO_5^+$] ($[M+H]^+$) 369.0960, found *m/z* 369.0966.

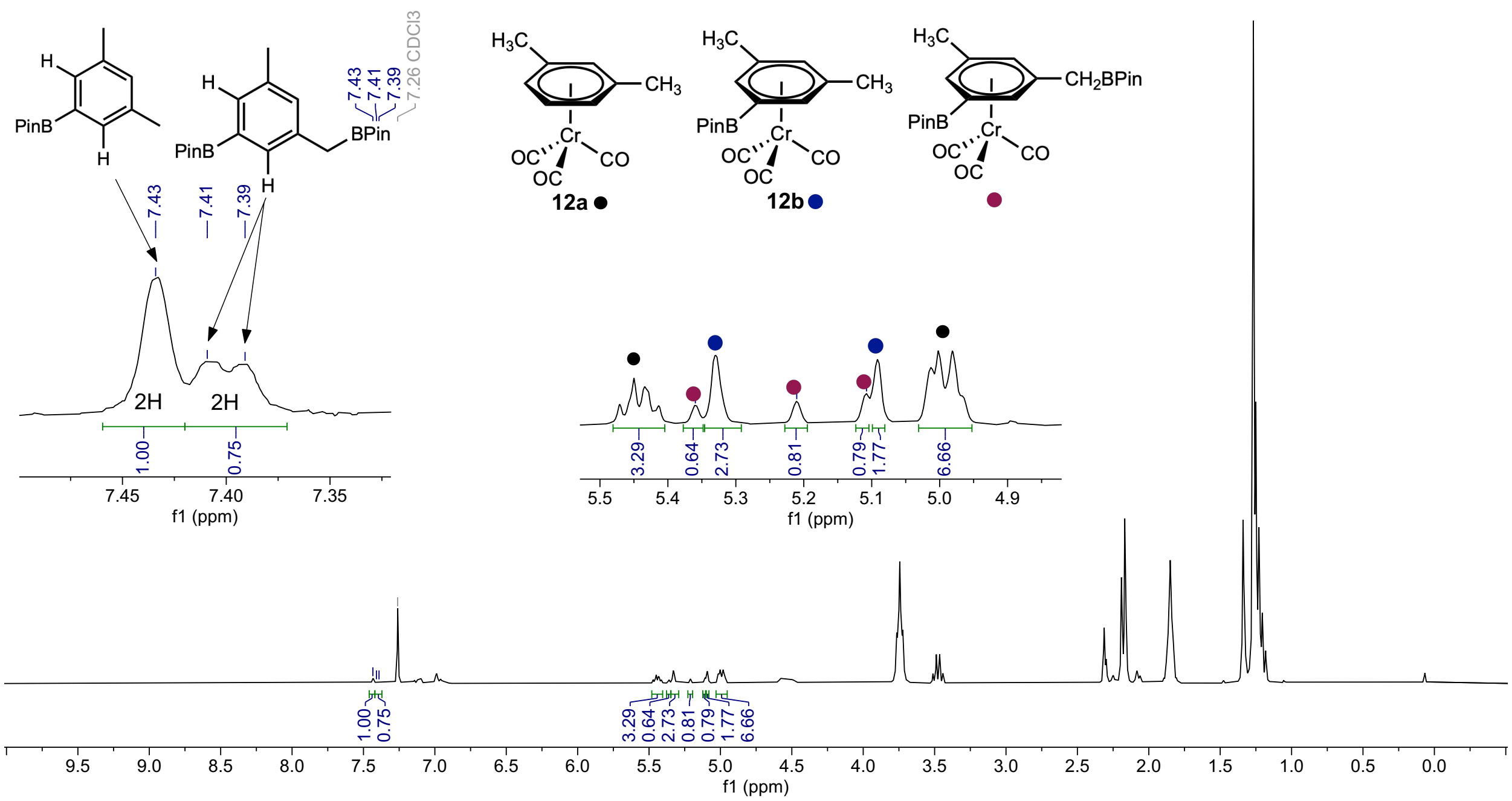

Figure S10. ^{1}H NMR spectrum (400 MHz, chloroform-*d*, 23 °C) of products resulting from borylation of **12a**.

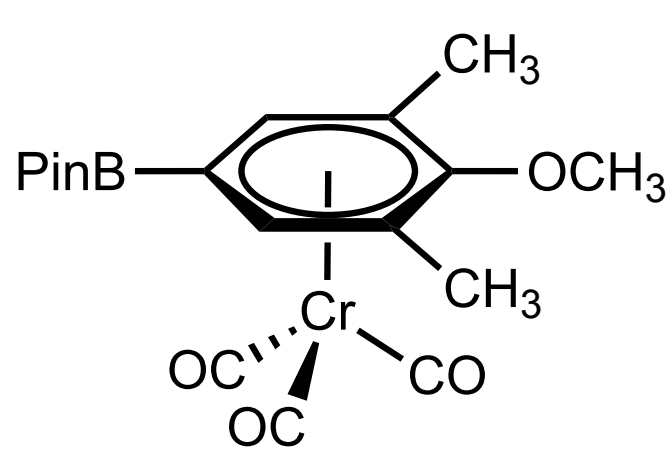

2-(4-Methoxy-3,5-dimethylphenyl)-4,4,5,5-Tetramethyl-1,3,2-dioxaborolane chromium tricarbonyl (13b) was prepared by adding 2,6-dimethylanisole chromium tricarbonyl (**13a**, 0.025 g, 0.085 mmol), B_2Pin_2 (0.025 g, 0.099 mmol), **5** (0.004 g), and THF (1 mL) to a vial containing a stir bar. The vial was capped, stirred and the solution turned brown. After 30 minutes, the flask was opened to air, 1,3,5-trimethoxybenzene (0.100 mL of a 50 mg/1.00 mL solution, 5 mg) was

added, an aliquot was removed, filtered through SiO_2 (diethyl ether) and the volatiles removed under a flow of dry nitrogen. Analytical data was consistent with literature values for the desired material and ^{1}H NMR showed a 41% yield of **13b**.

^{1}H NMR (400 MHz, acetone-d_6, 23 °C): δ 5.60 (s, 2H) , 3.78 (s, 3H), 2.28 (s, 6H), 1.33 (s, 12H).

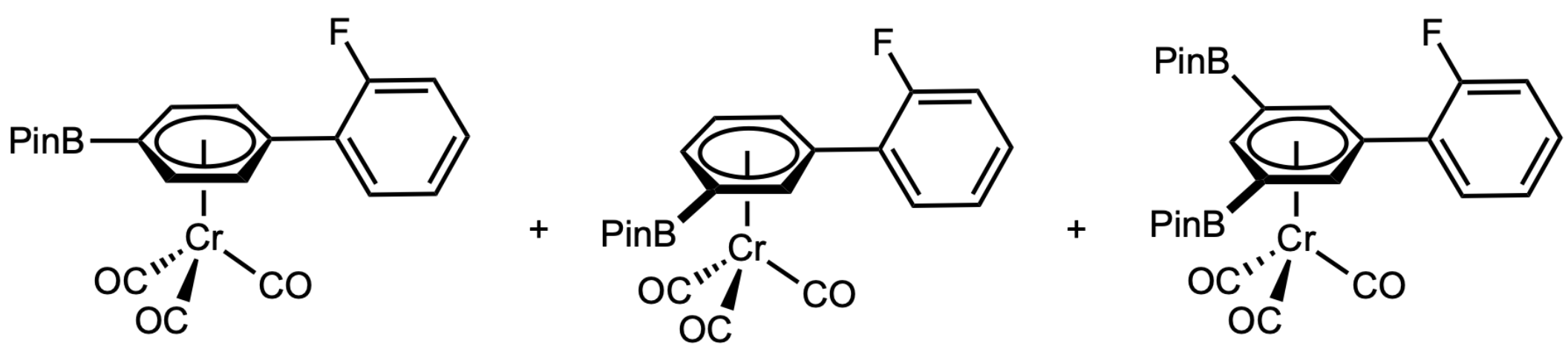

para-**14b** *meta*-**14b** *bis-meta*-**14b**

2-(2'-Fluoro-[1,1'-biphenyl]-3-yl)-4,4,5,5-tetramethyl-1,3,2-dioxaborolane chromium tricarbonyl (14b). Using the general procedure, to **14a** (0.032 g, 0.10 mmol) in THF (0.3 mL) was added 0.4 mL of a stock solution of B_2Pin_2 (0.75 M, 0.30 mmol) and **5** (0.014 M, 0.006 mmol). After 16 hours, the volatile components were removed under vacuum and 1,3,5-trimethoxybenzene (0.005 g) added. The material was chromatographed on SiO_2 (hexanes/diethyl ether) to give a 78% combined yield of a mixture of borylation isomers on the non-fluorinated ring, in a 19 (**14b** *meta*) : 37 (**14b** *para*) : 44 (**14b** *bis-meta*) ratio. Purification by chiral SFC with 18 % MeOH / 82% CO_2 (100 bar) as the mobile phase using a Phenomenex Lux® Amy-1 (250 x 30 mm) column (performed by Lotus Separations, LLC) resulted in the separation of these isomers (method: 85 mL/min; 218 nm). Competing decomposition in solution precluded efforts to obtain ^{13}C NMR spectra for some of the isomers.

<u>*meta*-**14b**:</u>

^{1}H NMR (400 MHz, chloroform-d, 23 °C): δ 7.37 – 7.06 (m, 4H, overlapping with decoordinated arene), 6.07 (p, J = 0.9 Hz, 1H), 5.93 (dq, J = 6.6, 1.3 Hz, 1H), 5.79 (dt, J = 6.3, 1.0 Hz, 1H), 5.31 (t, J = 6.4 Hz, 1H), 1.34 (s, 12H).

^{19}F NMR (376 MHz, chloroform-*d*, 23 °C): δ -115.38 – -115.55 (m), -118.00 (dt, *J* = 11.5, 5.7 Hz, decoordinated arene).

HRMS (ESI+) *m/z* calculated for [$C_{21}H_{20}BCrFO_5^+$] ($[M]^+$) 434.0793, found *m/z* 434.0792.

para-**14b**:

^{1}H NMR (400 MHz, chloroform-*d*, 23 °C): δ 7.34 (dq, *J* = 13.5, 7.0 Hz, 1H), 7.26 – 7.09 (m, 3H), 5.84 (d, *J* = 6.4 Hz, 1H), 5.60 (d, *J* = 6.5 Hz, 1H), 1.36 (s, 12H).

^{13}C NMR (101 MHz, chloroform-*d*, 23 °C): δ 232.28, 135.00, 130.91, 129.30, 128.51, 124.47, 25.03, 24.85.

^{19}F NMR (376 MHz, chloroform-*d*, 23 °C): δ -114.80.

HRMS (ESI+) *m/z* calculated for [$C_{21}H_{21}BCrFO_5^+$] ($[M+H]^+$) 435.0866, found *m/z* 435.0860.

bis-meta-**14b**:

^{1}H NMR (400 MHz, chloroform-*d*, 23 °C): δ 7.50 – 7.04 (m, 4H), 6.23 (t, *J* = 1.1 Hz, 2H), 6.16 (t, *J* = 1.0 Hz, 1H), 1.33 (s, 24H).

^{19}F NMR (376 MHz, chloroform-*d*, 23 °C): δ -115.25 (dt, *J* = 11.8, 5.5 Hz), -117.69 – -117.82 (m, decoordinated arene).

HRMS (ESI+) *m/z* calculated for [$C_{27}H_{31}B_2CrFO_7^+$] ($[M]^+$) 560.1645, found *m/z* 560.1661.

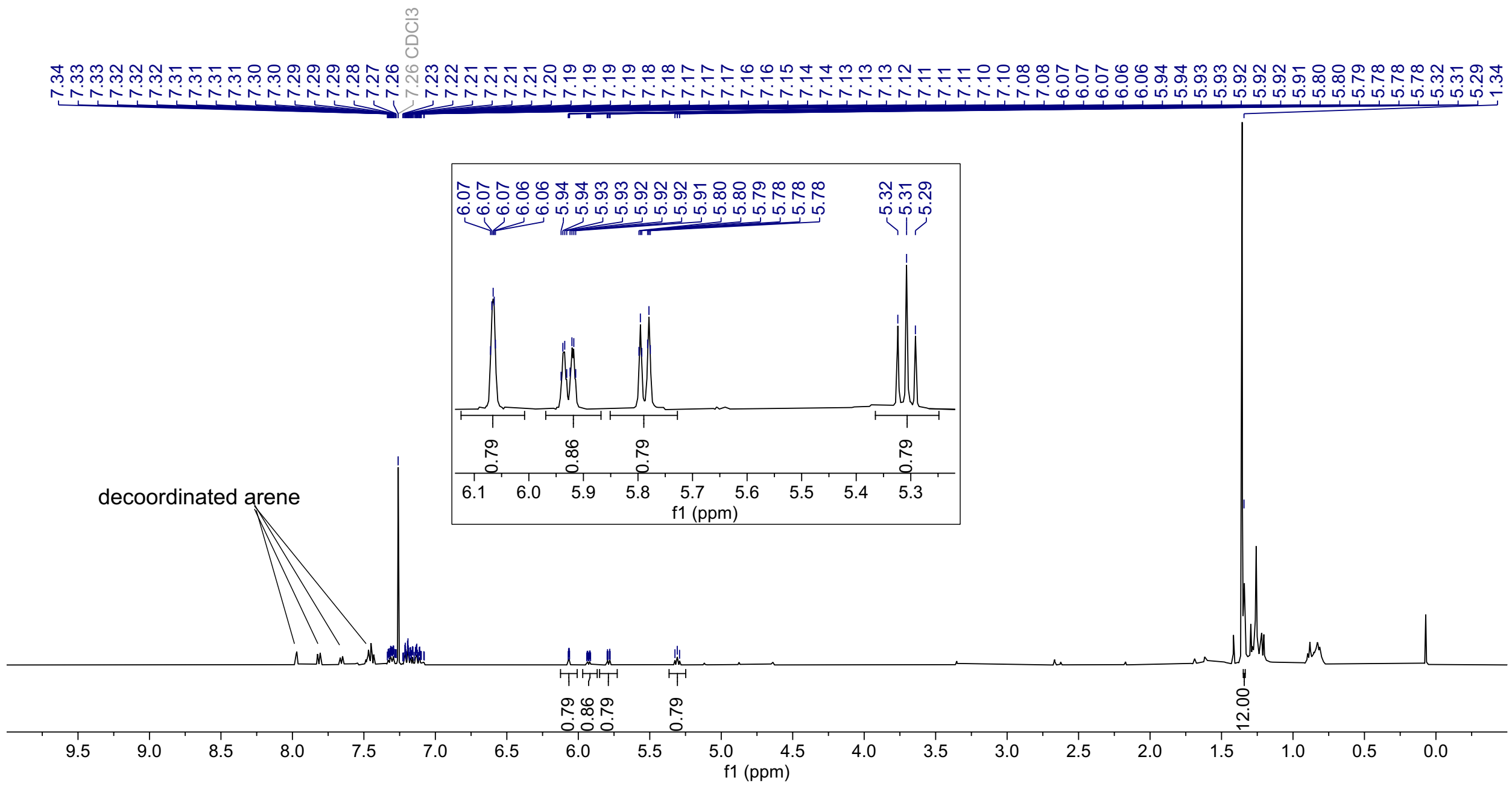

Figure S11. ^{1}H NMR spectrum (400 MHz, chloroform-*d*, 23 °C) of *meta*-**14b**.

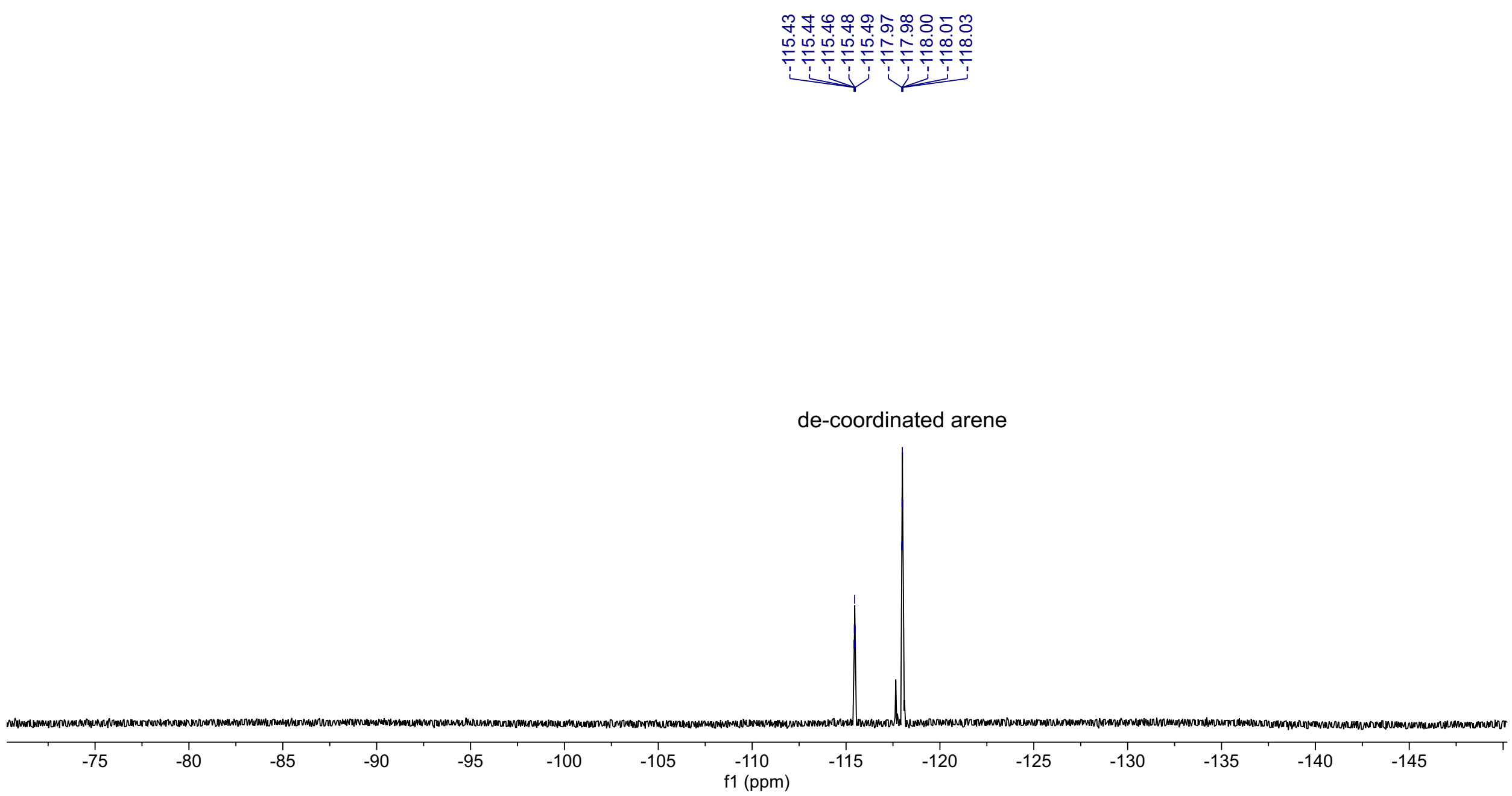

Figure S12. ^{19}F NMR spectrum (376 MHz, chloroform-d, 23 °C) of *meta*-**14b**.

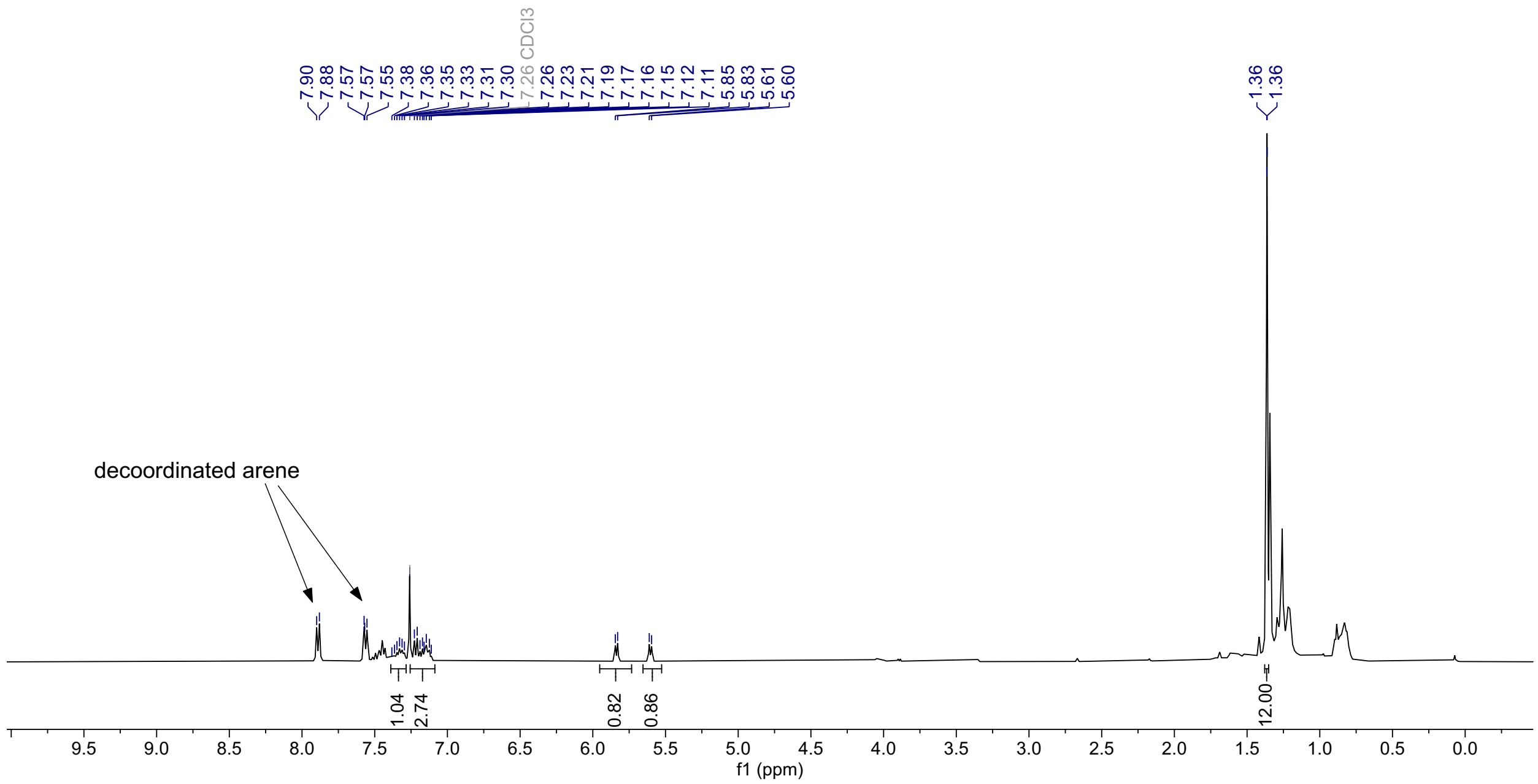

Figure S13. ^{1}H NMR spectrum (400 MHz, chloroform-*d*, 23 °C) of *para*-**14b**.

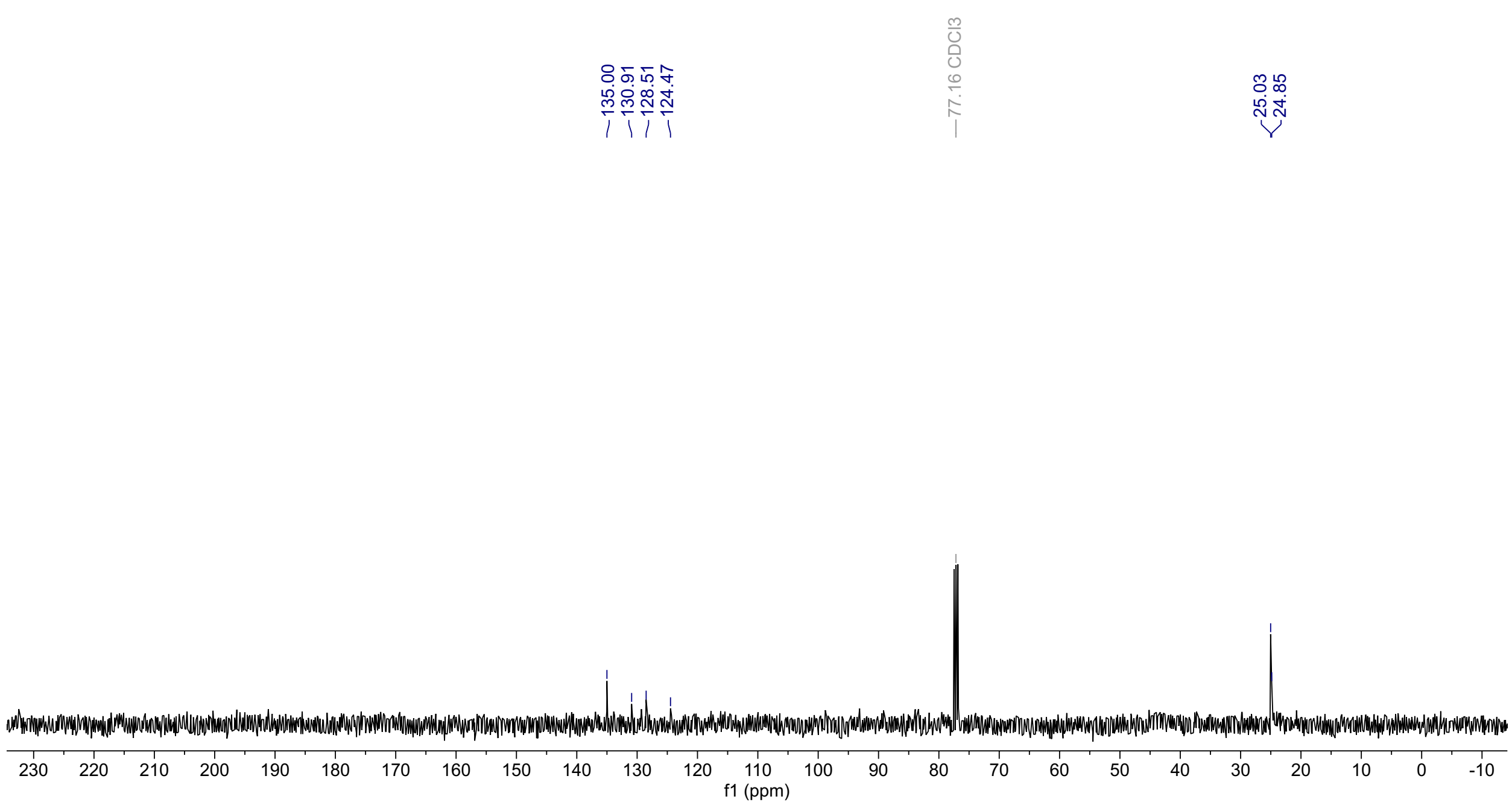

Figure S14. ^{13}C NMR spectrum (101 MHz, chloroform-*d*, 23 °C) of *para*-**14b**.

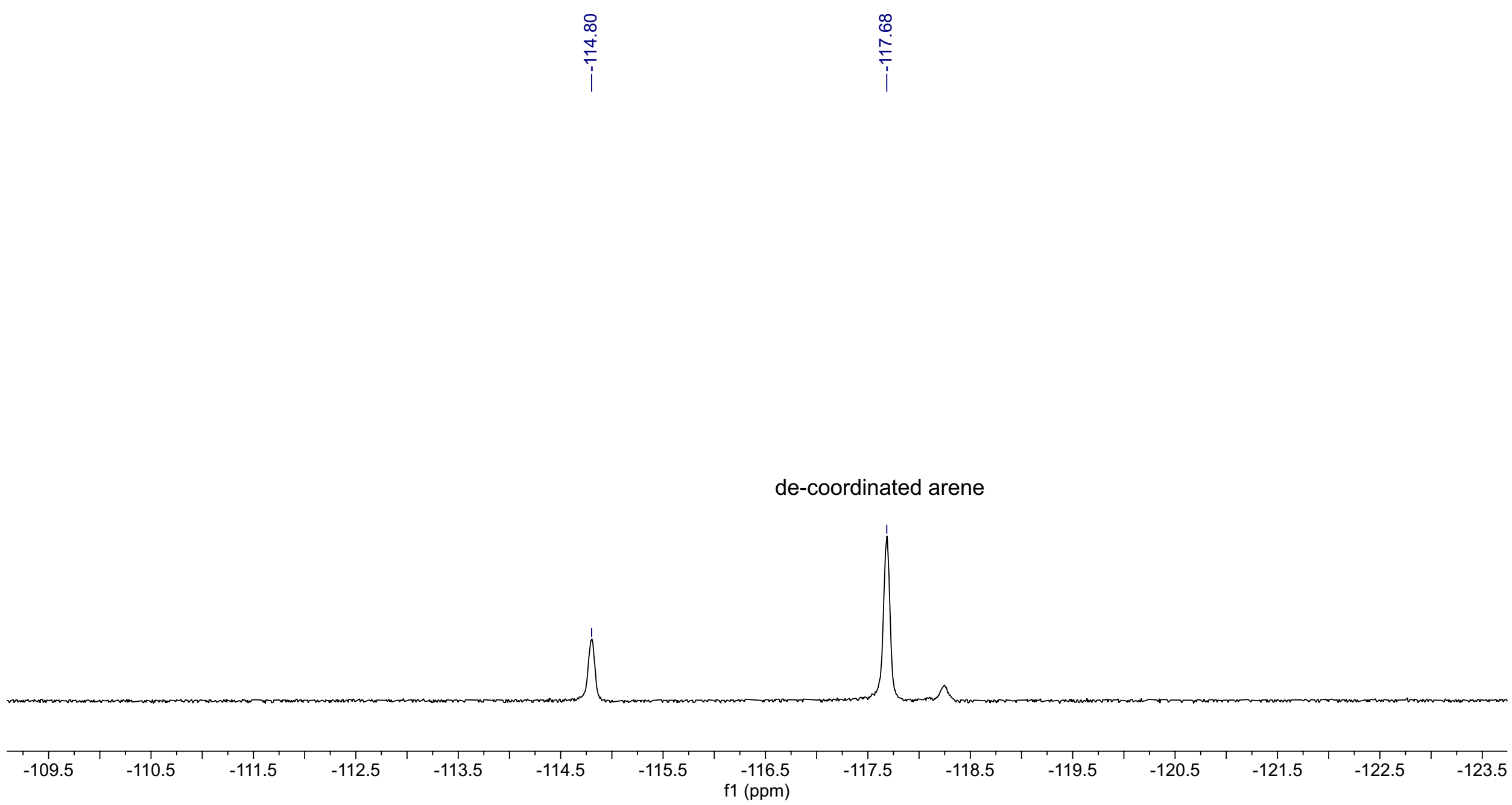

Figure S15. ^{19}F NMR spectrum (376 MHz, chloroform-d, 23 °C) of *para*-**14b**.

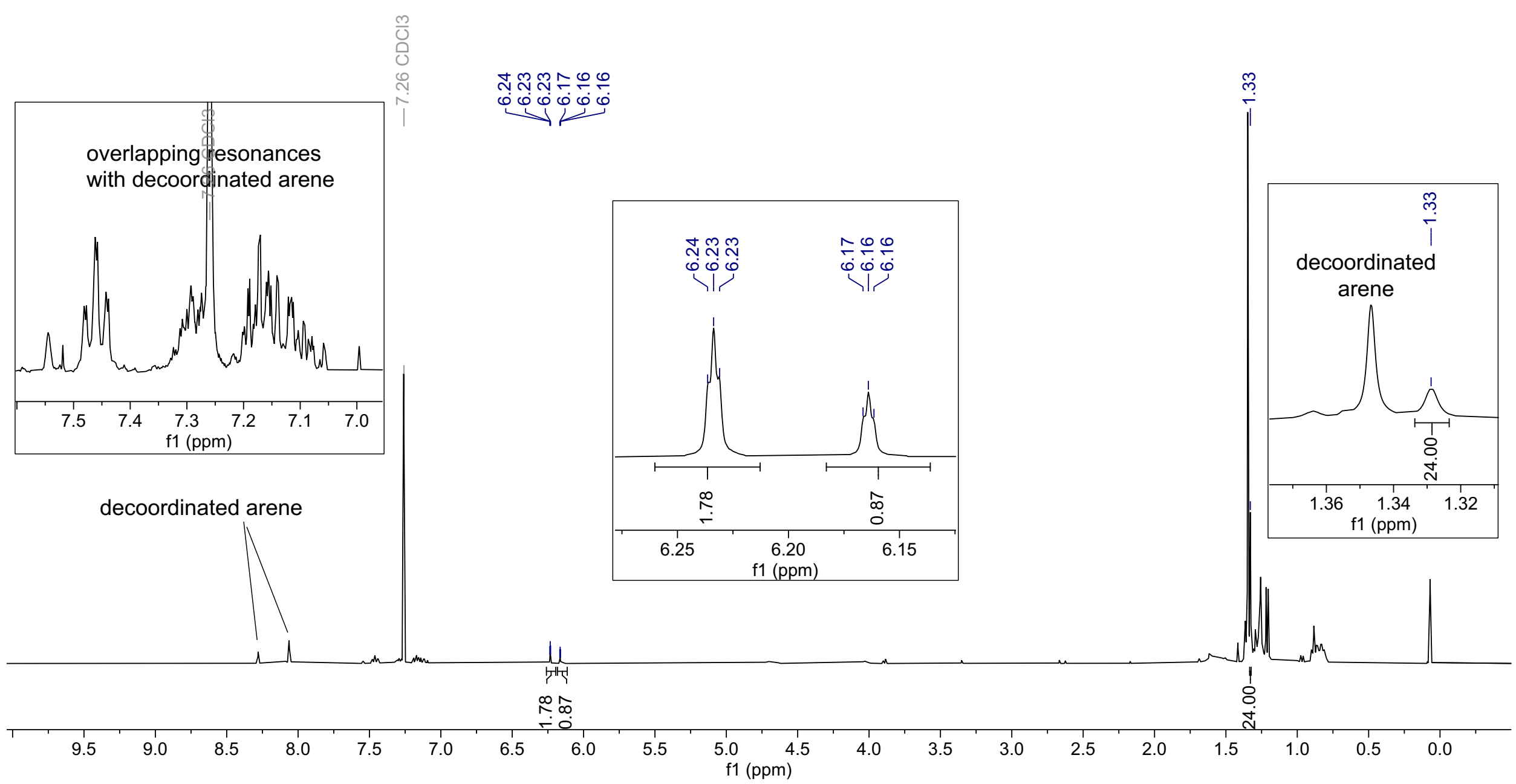

Figure S16. ^{1}H NMR spectrum (400 MHz, chloroform-*d*, 23 °C) of *bis-meta*-**14b**.

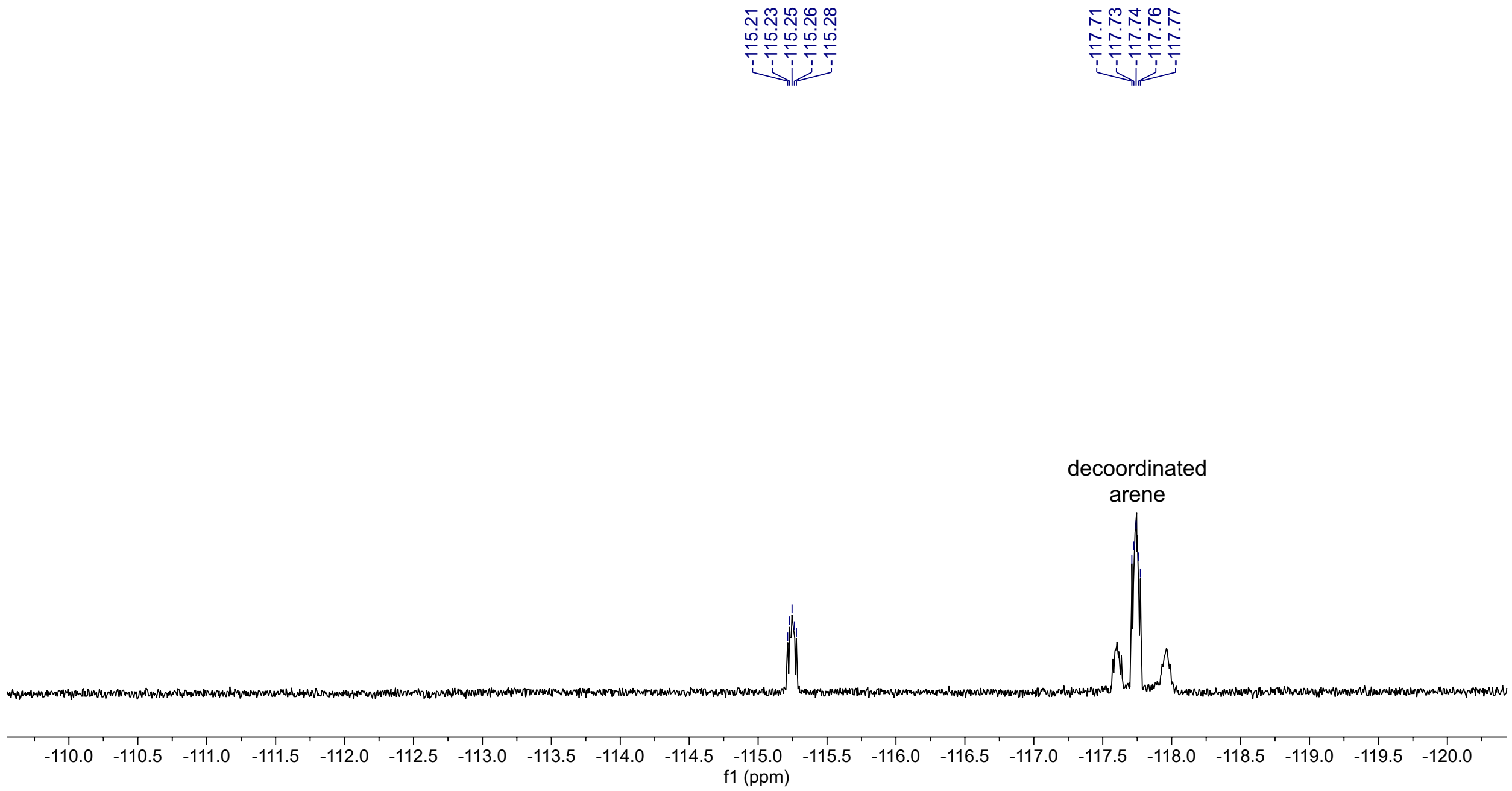

Figure S17. ^{19}F NMR spectrum (376 MHz, chloroform-*d*, 23 °C) of *bis-meta*-**14b**.

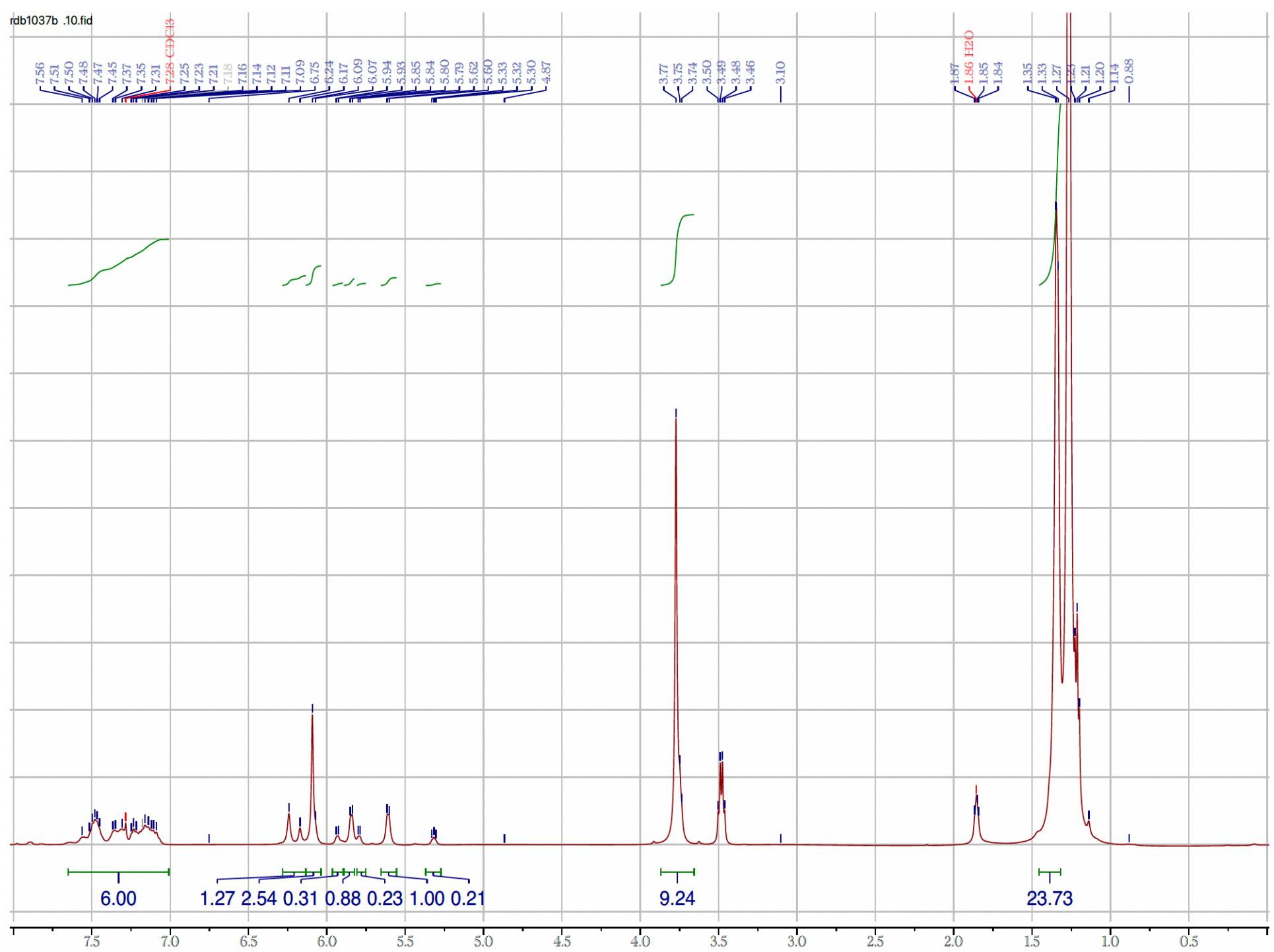

Figure S18. ^{1}H NMR spectrum (400 MHz, chloroform-*d*, 23 °C) of products resulting from borylation of **14b**.

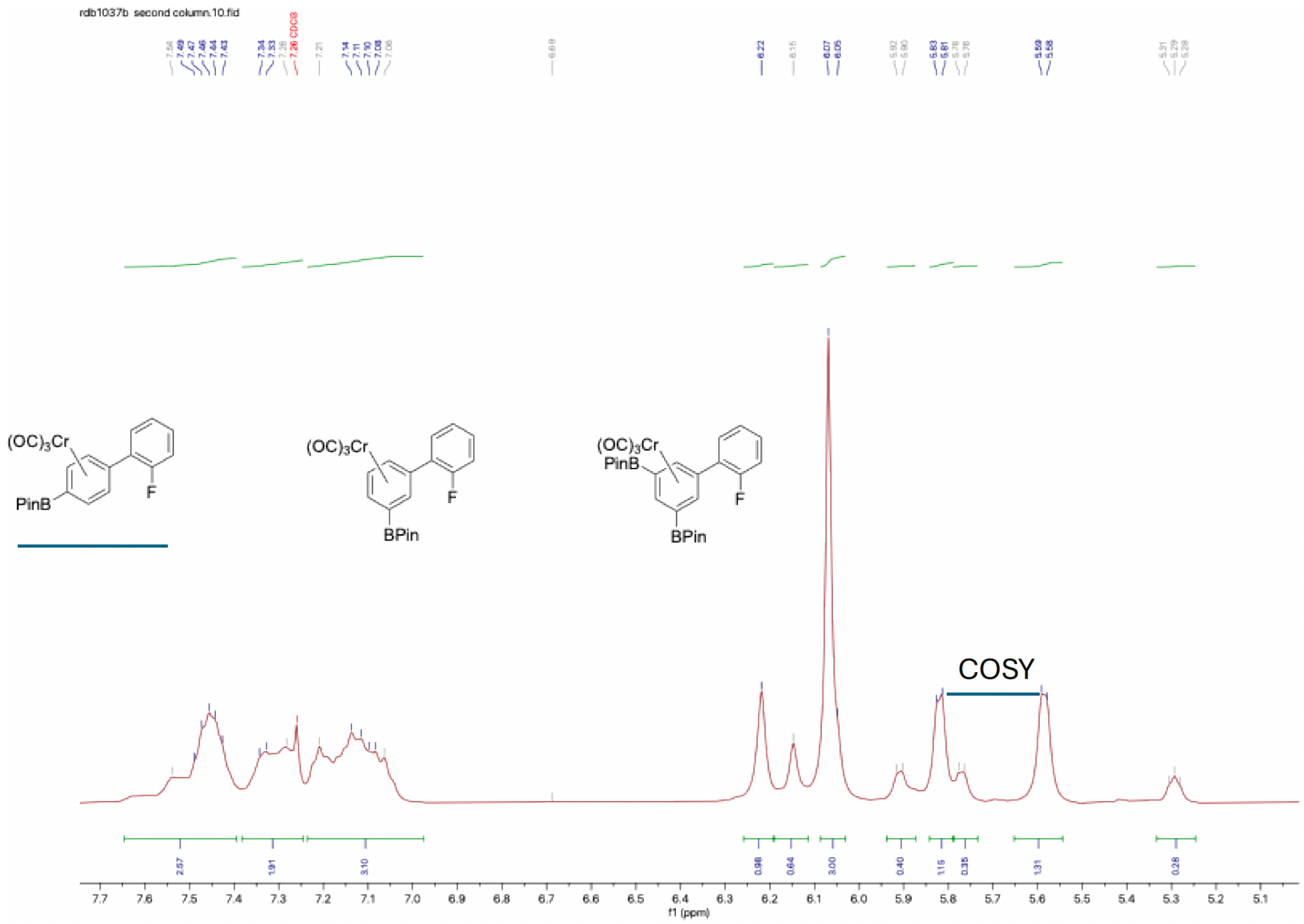

Figure S19. ^{1}H NMR spectrum (400 MHz, chloroform-*d*, 23 °C) showing the assignment of product *para*-**14b** based on ^{1}H-^{1}H COSY correlation.

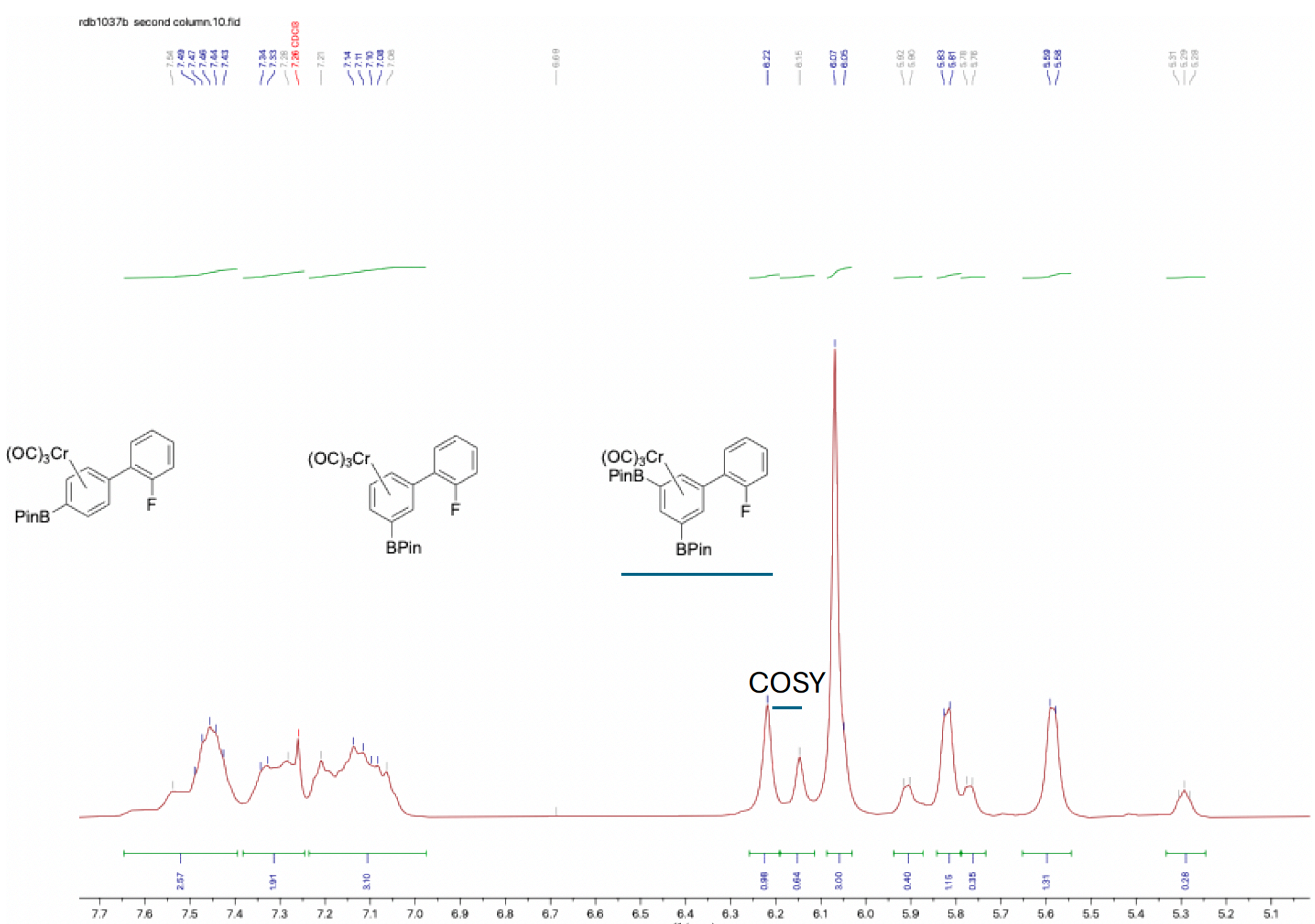

Figure S20. ^{1}H NMR spectrum (400 MHz, chloroform-*d*, 23 °C) showing the assignment of product *bis-meta*-***14b*** based on ^{1}H-^{1}H COSY correlation.

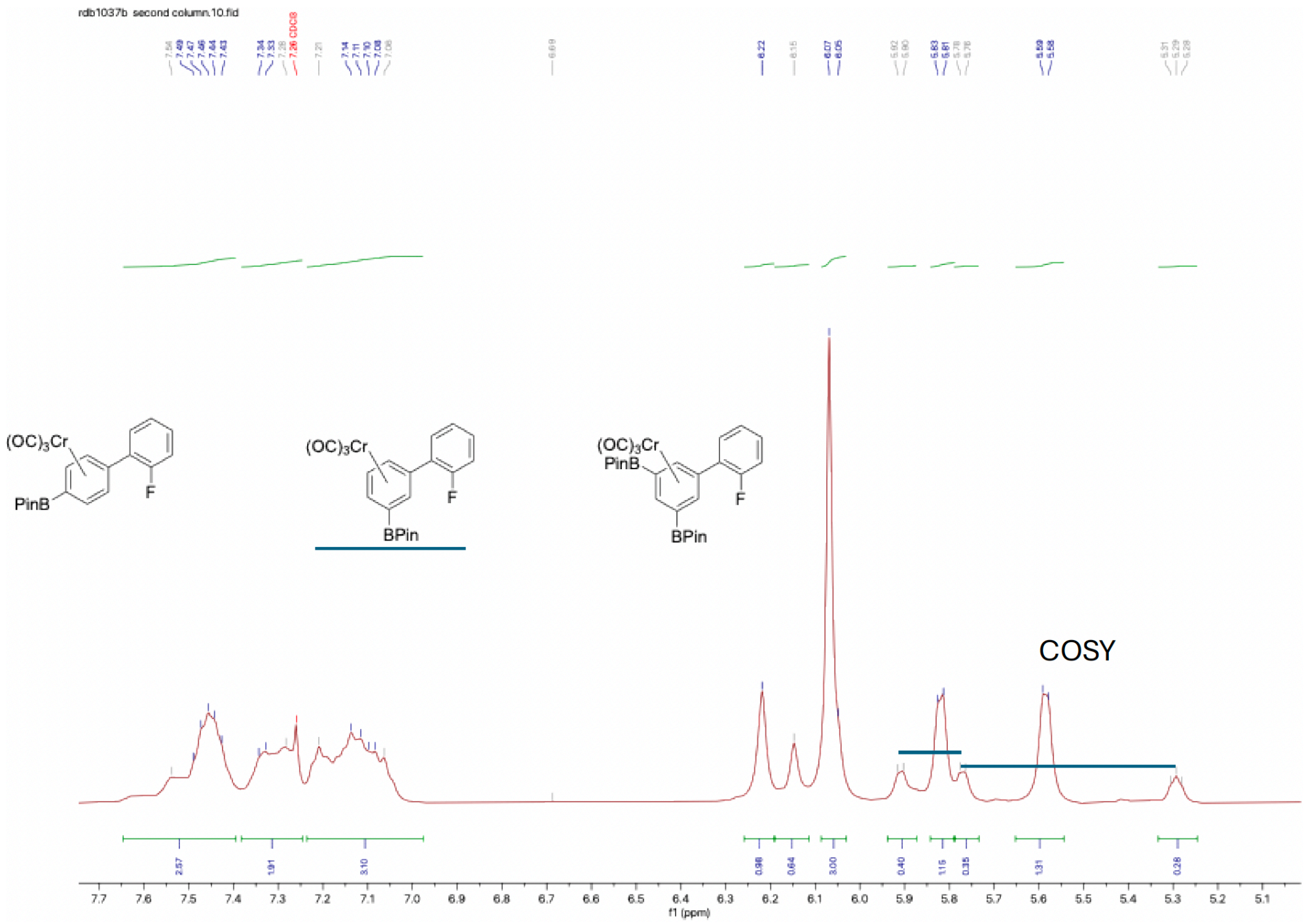

Figure S21. ^{1}H NMR spectrum (400 MHz, chloroform-*d*, 23 °C) showing the assignment of product *meta-**14b*** based on ^{1}H-^{1}H COSY correlation.

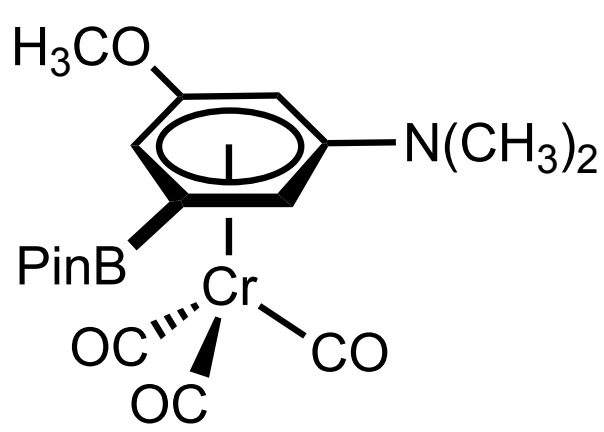

3-Methoxy-*N*,*N*-dimethyl-5-(4,4,5,5-tetramethyl-1,3,2-dioxaborolan-2-yl)aniline chromium tricarbonyl (**16b**) was prepared by the addition of 3-methoxy-*N*,*N*-dimethylaniline chromium tricarbonyl (**16a**, 0.014 g, 0.049 mmol), B_2Pin_2 (0.014 g, 0.055 mmol), **5** (0.0035 g, 0.005 mmol) and THF (0.5 mL) to a vial containing a stir bar. The vessel was capped and allowed to stir and the color changed to brown. After 23 hours, the reaction was removed from the glovebox, and 1,3,5-trimethoxybenzene was added (2.9 mg). The mixture was filtered through SiO_2 ($CHCl_3$), and the volatiles were removed by rotary evaporation. Analytical data was consistent with literature values for the desired material and ^{1}H NMR showed an 88% NMR yield of **16b**.[12]

^{1}H NMR (400 MHz, acetone-d_6, 23 °C): δ 5.25 (t, *J* = 2.2 Hz, 1H), 5.02 (d, *J* = 2.1 Hz, 1H), 4.77 (d, *J* = 2.2 Hz, 1H), 3.80 (s, 3H), 2.99 (s, 6H), 1.38 (s, 12H).

V. Competition Experiment

Separate-Flask Competition Experiment

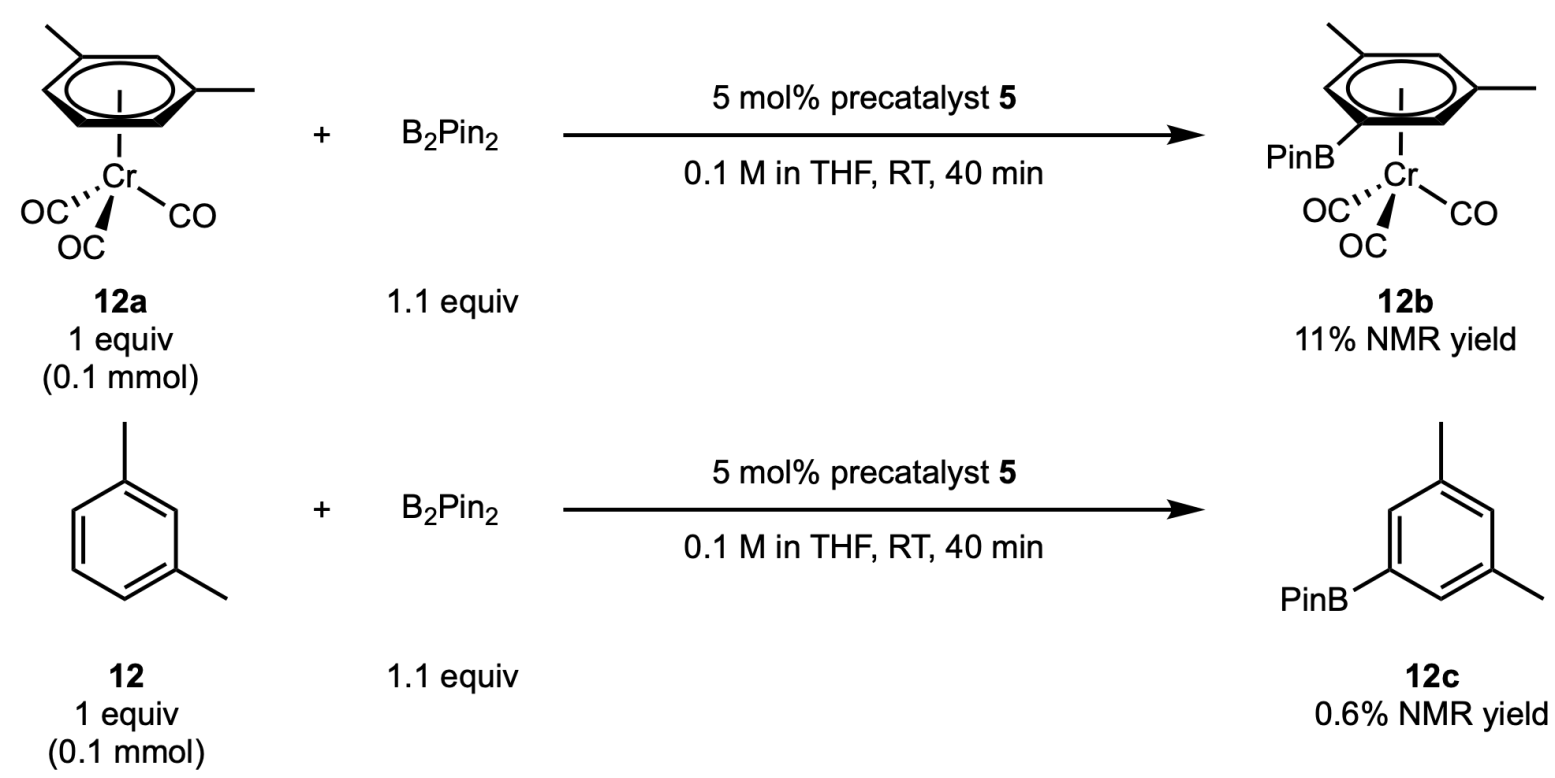

In two 1-dram vials containing a stir bar each, 1,3-dimethylbenzene chromium tricarbonyl (**12a**, 24 mg, 0.1 mmol) and *m*-xylene (**12,** 11 mg, 0.1 mmol) were weighed out separately. To each vial, 1.0 mL of THF stock solution containing B_2Pin_2 (56 mg, 0.22 mmol) and **5** (6 mg, 0.01 mmol) were added simultaneously. The vials were then capped, stirred and the solution turned deep brown. After 40 min, the vials were transferred outside of the glovebox, exposed to air and the volatiles evaporated. Following filtration through SiO_2 (diethyl ether), evaporation of the solvent, and addition of a stock solution of 1,3,5-trimethoxybenzene as an internal standard, quantitative ^{1}H NMR spectra (**Figure S22** and **Figure S23**) showed an approximately 20 : 1 ratio of **12b** to **12c**.

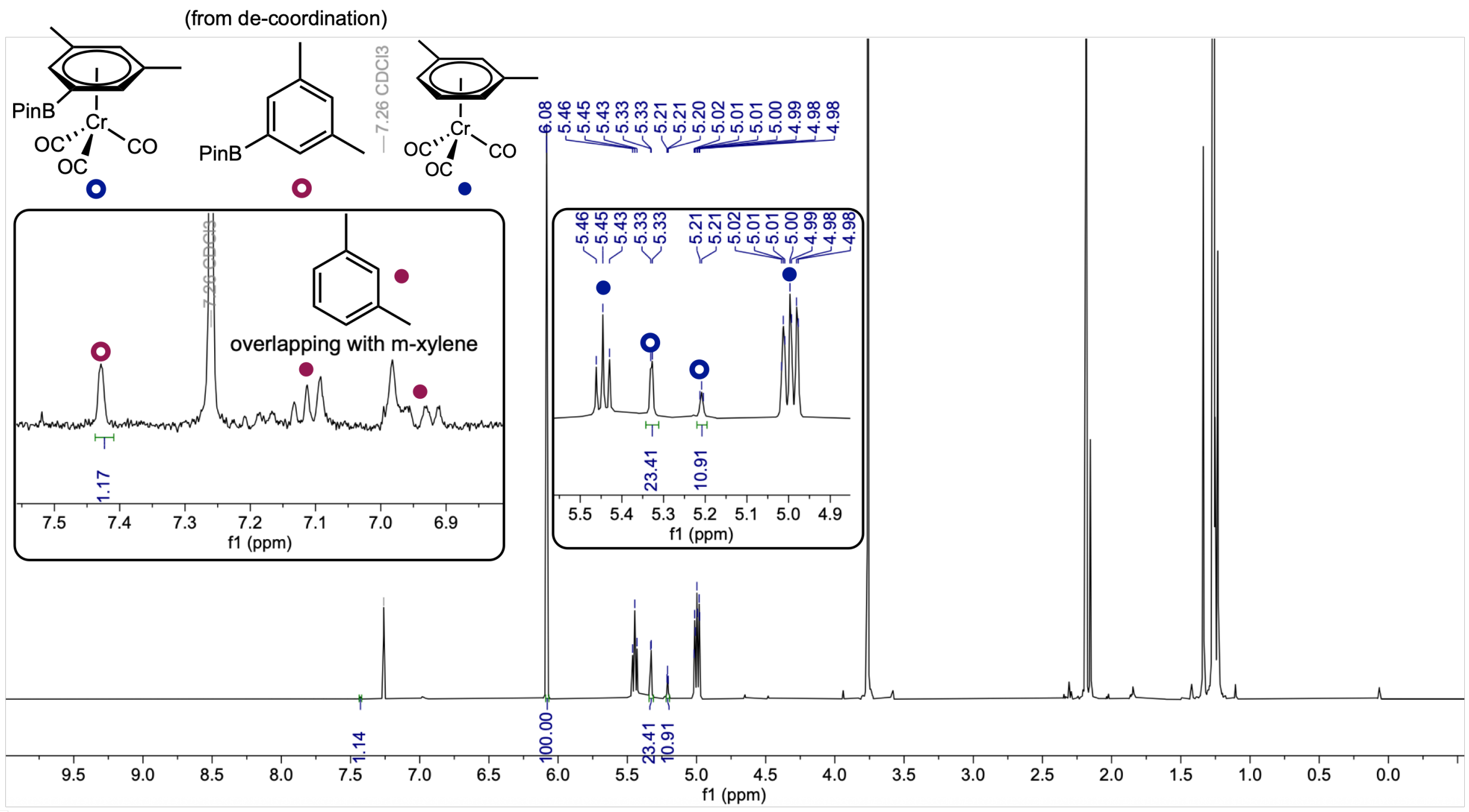

Figure S22. ^{1}H NMR spectrum (400 MHz, chloroform-*d*, 23 °C) of crude reaction mixture resulting from borylation of **12a** after 40 minutes at room temperature.

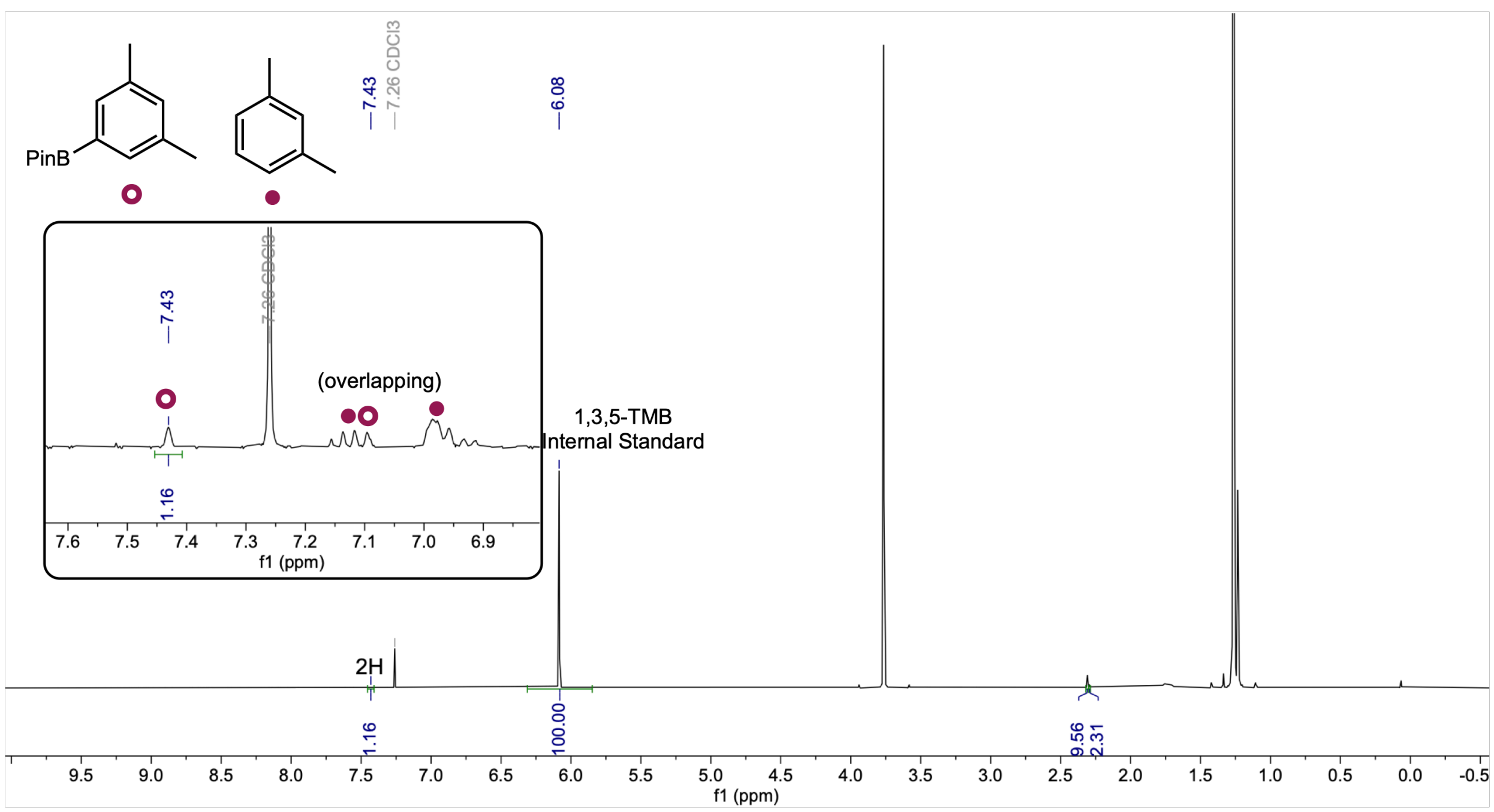

Figure S23. ^{1}H NMR spectrum (400 MHz, chloroform-*d*, 23 °C) of crude reaction mixture resulting from borylation of **12** after 40 minutes at room temperature.

Same-Flask Competition Experiment

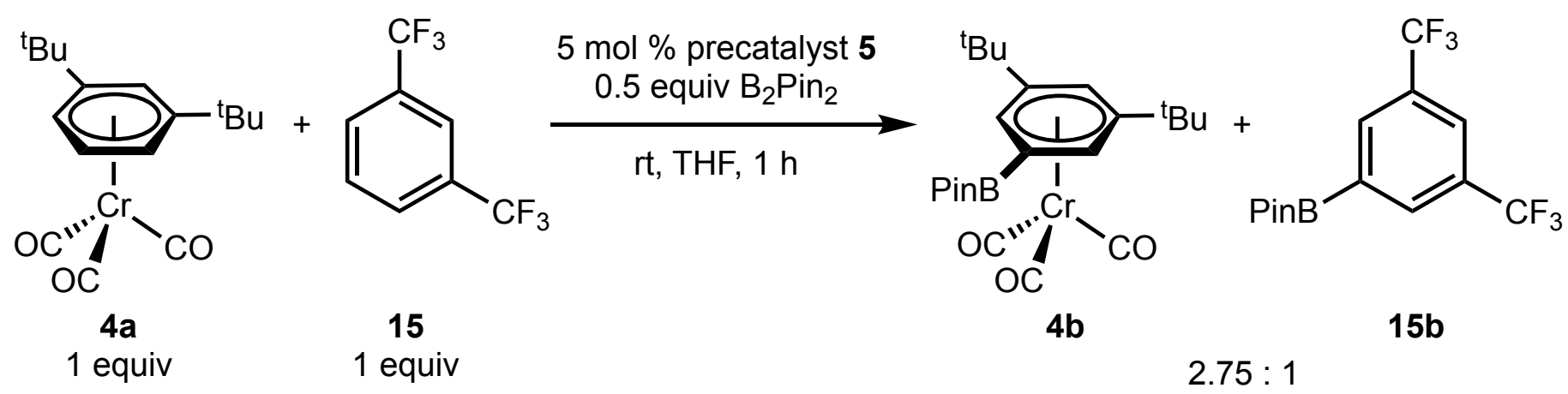

1,3-di-*tert*-butylphenyl chromium tricarbonyl (**4a**, 0.16 g, 0.049 mmol), 1,3-bis(trifluoromethyl)benzene (**15,** 5 µL, 0.052 mmol), B_2Pin_2 (0.013 g, 0.051 mmol), **5** (0.10 mL, 0.047 M in THF, 0.0047 mmol), and THF (0.5 mL) were added to a vial containing a stir bar. The vial was capped, stirred and the solution turned light brown. After 1 hour, the reaction was stopped and the volatiles evaporated. Following filtration through SiO_2 (diethyl ether) and evaporation of the solvent, quantitative ^{1}H NMR showed a 1.8 : 1 ratio of **4b** to **15b**.

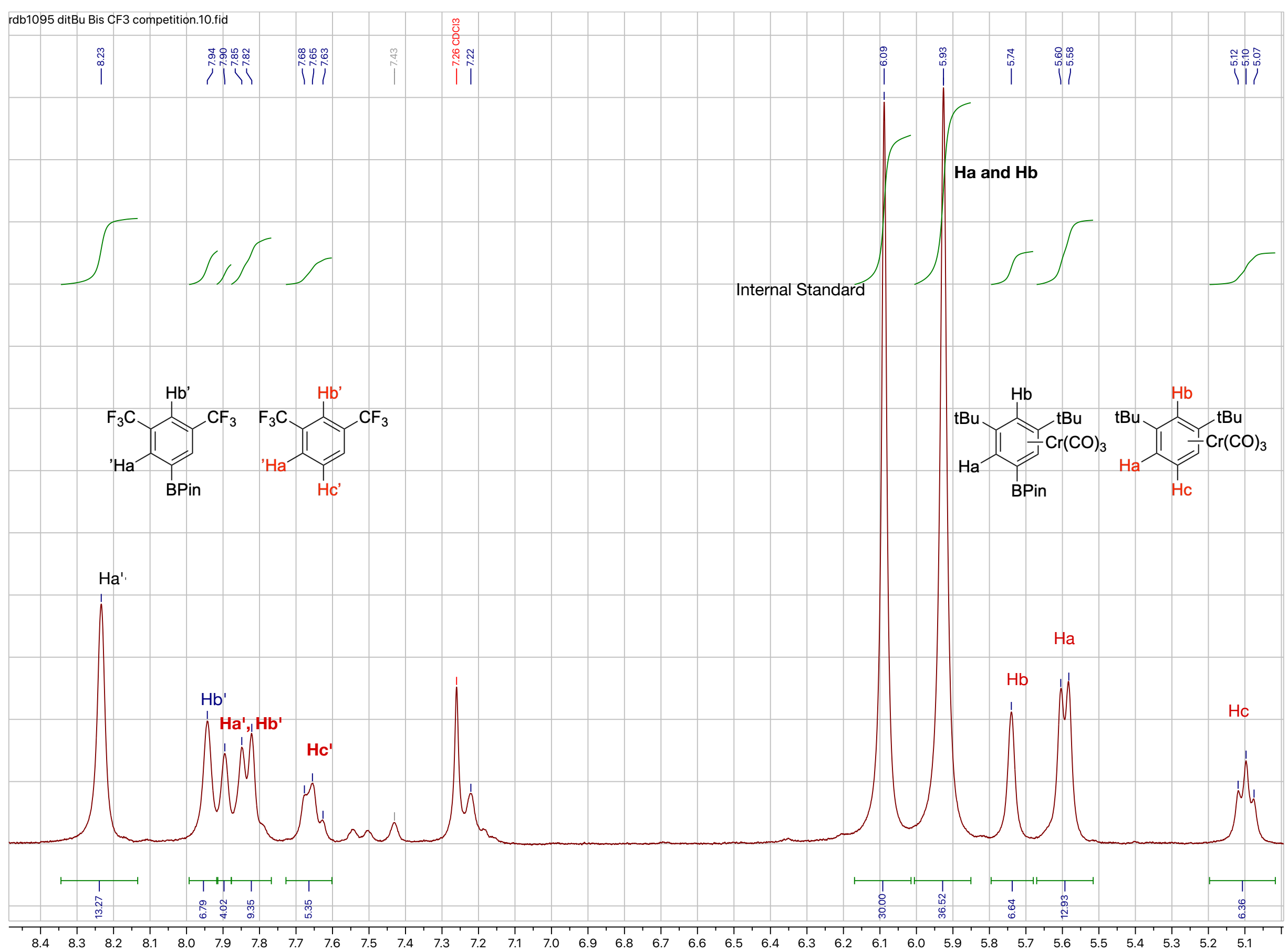

Figure S24. ^{1}H NMR spectrum (400 MHz, chloroform-*d*, 23 °C) reflecting the outcomes of the same-vessel competition experiment between **4a** and **15**.

VI. Challenging Substrates

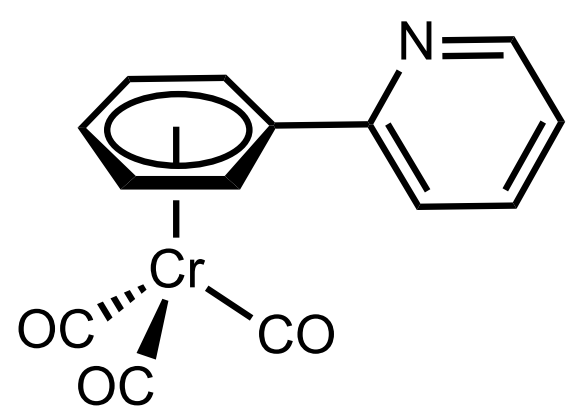

(2-pyridyl)benzene chromium tricarbonyl (0.029 g, 0.10 mmol, 1.0 equiv), B_2Pin_2 (0.028 g, 0.11 mmol, 1.1 equiv) and **5** (3 mg, 0.005 mmol) and THF (1.0 mL) were added to a vial with a stir bar. We note that **5** was added as a 0.5 mL stock solution in THF, prepared by dissolving 15 mg of **5** in 2.5 mL of THF. The color turned deep orange brown. After 48 hours, the reaction was opened to atmosphere, 1,3,5-trimethoxybenzene (5 mg) added and the solution filtered through a short plug of SiO_2 (THF). The volatiles were evaporated under a stream of dry nitrogen. Quantitative ^{1}H NMR indicated no conversion/borylation. In a separate experiment, pre-stirring **5** and B_2Pin_2 for 1 hour at room temperature prior to the addition to (2-pyridyl)benzene chromium tricarbonyl led to minimal conversion as judged by quantitative ^{1}H NMR.

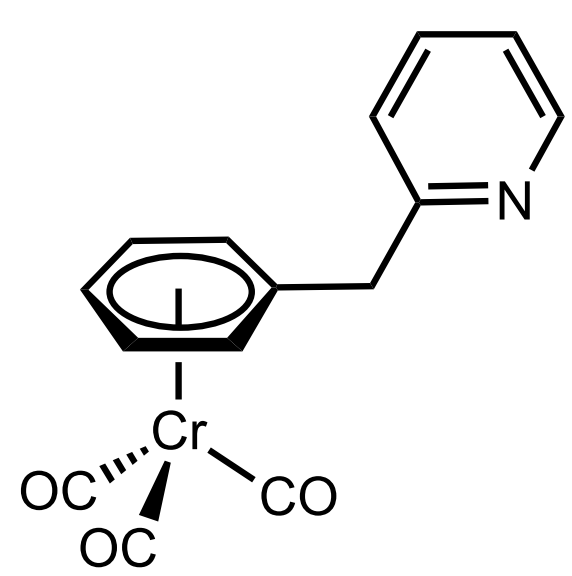

(2-pyridylmethyl)benzene chromium tricarbonyl (0.029 g, 0.094 mmol), B_2Pin_2 (0.026 g, 0.10 mmol) and **5** (0.2 mL, 0.022 M in THF) and THF (0.8 mL) were added to a vial with a stir bar. The color turned green/brown. After 48 hours, the reaction was opened to atmosphere, 1,3,5-trimethoxybenzene (5 mg) added and the solution filtered through a short plug of SiO_2 (THF). The volatile materials were evaporated under a stream of dry nitrogen. NMR showed no evidence for borylation.

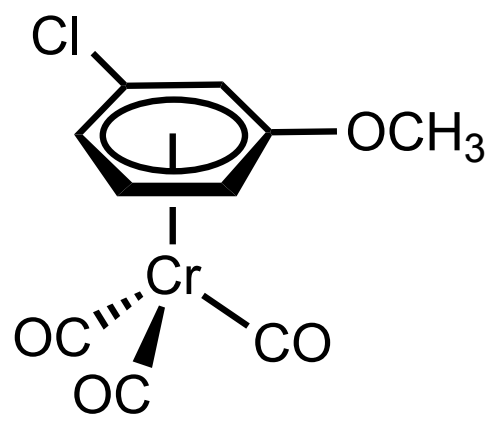

3-chloroanisole chromium tricarbonyl (0.024 g, 0.087 mmol), B_2Pin_2 (0.4 mL, 2 M in THF, 0.08 mmol), **5** (0.1 mL, 0.047 M in THF), 1,3,5-trimethoxybenzene (0.2 mL, 0.16 M in THF) and THF (0.3 mL) were added to a vial with a stir bar. The color did not change. Aliquots removed at 2, 24, and 72 hours showed no evidence of borylation. Some chromium-free aromatic resonances were observed.

VII. References

1. Pangborn, A. B.; Giardello, M. A.; Grubbs, R. H.; Rosen, R. K.; Timmers, F. J. Safe and Convenient Procedure for Solvent Purification. *Organometallics* **1996**, *15*, 1518–1520.

2. Shimozono, A. M.; Li, H.; Bisht, S.; Khatri, B.; Chen, L.; Xu, D.; Pecoraro, M. V.; Roque, J. B.; Salter, R.; Shatruk, M.; Lin, R.; Chirik, P. J. Alkoxide-Bridged Dicobalt(II) Compounds as Precatalysts for C–H Borylation and Application to Late-Stage Functionalization. *ACS Catal.* **2026**, *16*, 9238–9246.

3. Anson, C. E.; Klopper, W.; Li, J.-S.; Ponikiewski, L.; Rothenberger, A. A Close Look at Short C–CH_3···Potassium Contacts: Synthetic and Theoretical Investigations of [$M_2Co_2(\mu_3$-$O^tBu)_2(\mu_2$-$O^tBu)_4(THF)_n$] (M=Nsa, K, Rb, THF=tetrahydrofuran). *Chem. Eur. J.* **2006**, *12*, 2032–2038.

4. Roque, J. B.; Shimozono, A. M.; Pabst, T. P.; Hierlmeier, G.; Peterson, P. O.; Chirik, P. J. Kinetic and Thermodynamic Control of C(sp^2)–H Activation Enables Site-Selective Borylation. *Science* **2023**, *382*, 1165–1170.

5. Fulmer, G. R.; Miller, A. J. M.; Sherden, N. H.; Gottlieb, H. E.; Nudelman, A.; Stoltz, B. M.; Bercaw, J. E.; Goldberg, K. I. NMR Chemical Shifts of Trace Impurities: Common Laboratory Solvents, Organics, and Gases in Deuterated Solvents Relevant to the Organometallic Chemist. *Organometallics* **2010**, *29*, 2176–2179.

6. Ricci, P.; Krämer, K.; Larrosa, I. Tuning Reactivity and Site Selectivity of Simple Arenes in C–H Activation: Ortho-Arylation of Anisoles via Arene–Metal π-Complexation. *J. Am. Chem. Soc.* **2014**, *136*, 18082–18086.

7. Maurer, C.; Fleischer, R.; Mandal, A.; Chandramohan, K. R. K.; Herpell, F. C.; Köhler, C. H.; Cetin, D.; Schnakenburg, G.; Bunescu, A. Extensive Studies on the Synthesis and Characterization of π-Arene Chromium Complexes and Their Performance in S_NAr and Suzuki–Miyaura Cross-Coupling Reactions. *Chem. Asian J.* **2025**, *20*, e202500139.

8. Mahaffy, C. A. L. The Synthesis and Characterization of Tricarbonylchromium Complexes of Disubstituted Anilines and Fluorobenzenes. *J. Organomet. Chem.* **1984**, *262*, 33-37.

9. Hijazi, A.; Djukic, J.-P.; Allouche, L.; de Cian, A.; Pfeffer, M.; Le Goff, X.-F.; Ricard, L. Stereoselective Cyclometalation of Planar Pro-chiral (η^6-Arene)tricarbonylchromium Complexes with OC-6-[$Ru(CO)_2Cl_2]_n$. *Organometallics* **2007**, *26*, 4180-4196.

10. Duke, C. B., III; Letterman, R. G.; Johnson, J. O.; Barr, J. W.; Hu, S.; Ross, C. R., II; Webster, C. E.; Burkey, T. J. Photochemistry of Chromium Arene Tricarbonyl Complexes with Tethered Pyridinyl and Propenyl Groups: Investigations of the Effect of Ring Size on Chelate Formation, Structure, and Linkage Isomerization. *Organometallics* **2014**, *33*, 485-497.

11. Shimozono, A. M.; Roque, J. B.; Li, H.; Zhang, T.; Lin, R.; Chirik, P. J. Alkene Borylation–Hydrogenation Enables Highly Active, Site-Selective Cobalt-Catalyzed Borylation. *J. Am. Chem. Soc.* **2025**, *147*, 26437–26445.

12. Mandal, A.; Maurer, C.; Plett, C.; Chandramohan, K. R. K.; Fleischer, R.; Schnakenburg, G.; Grimme, S.; Bunescu, A. Selective C–H Borylation of Polyaromatic Compounds Enabled by Metal-Arene π-Complexation. *J. Am. Chem. Soc.* **2025**, *147*, 15281–15293.
